# Sustained signalling by PTH modulates IP_3_ accumulation and IP_3_ receptors through cyclic AMP junctions

**DOI:** 10.1242/jcs.163071

**Published:** 2015-01-15

**Authors:** Abha Meena, Stephen C. Tovey, Colin W. Taylor

**Affiliations:** Department of Pharmacology, University of Cambridge, Cambridge, CB2 1PD, UK

**Keywords:** Ca^2+^ signalling, Cyclic AMP, Inositol trisphosphate receptor, Parathyroid hormone

## Abstract

Parathyroid hormone (PTH) stimulates adenylyl cyclase through type 1 PTH receptors (PTH_1_R) and potentiates the Ca^2+^ signals evoked by carbachol, which stimulates formation of inositol 1,4,5-trisphosphate (IP_3_). We confirmed that in HEK cells expressing PTH_1_R, acute stimulation with PTH(1-34) potentiated carbachol-evoked Ca^2+^ release. This was mediated by locally delivered cyclic AMP (cAMP), but unaffected by inhibition of protein kinase A (PKA), exchange proteins activated by cAMP, cAMP phosphodiesterases (PDEs) or substantial inhibition of adenylyl cyclase. Sustained stimulation with PTH(1-34) causes internalization of PTH_1_R–adenylyl cyclase signalling complexes, but the consequences for delivery of cAMP to IP_3_R within cAMP signalling junctions are unknown. Here, we show that sustained stimulation with PTH(1-34) or with PTH analogues that do not evoke receptor internalization reduced the potentiated Ca^2+^ signals and attenuated carbachol-evoked increases in cytosolic IP_3_. Similar results were obtained after sustained stimulation with NKH477 to directly activate adenylyl cyclase, or with the membrane-permeant analogue of cAMP, 8-Br-cAMP. These responses were independent of PKA and unaffected by substantial inhibition of adenylyl cyclase. During prolonged stimulation with PTH(1-34), hyperactive cAMP signalling junctions, within which cAMP is delivered directly and at saturating concentrations to its targets, mediate sensitization of IP_3_R and a more slowly developing inhibition of IP_3_ accumulation.

## INTRODUCTION

Parathyroid hormone (PTH) is the major endocrine regulator of plasma Ca^2+^ and phosphate concentrations and, with PTH-related peptide (PTHrP), it regulates bone remodelling (Potts and Gardella, 2007). Many effects of PTH and PTHrP are mediated by type 1 PTH receptors (PTH_1_Rs), which are G-protein-coupled receptors (GPCRs) (Mahon, 2012; Mannstadt et al., 1999). PTH receptors, along with other class II GPCRs, stimulate both adenylyl cyclase activity and an increase in the cytosolic free Ca^2+^ concentration ([Ca^2+^]_c_) (Taylor and Tovey, 2012). The N-terminal fragments of PTH and PTHrP, PTH(1-34) and PTHrP(1-36), are sufficient for activation of PTH_1_R (Mahon, 2012). However, PTH analogues differ in whether they favour PTH_1_R coupling to G proteins or other signalling proteins, notably GPCR kinases and β-arrestins (Dean et al., 2008; Gesty-Palmer and Luttrell, 2011; Okazaki et al., 2008). Binding of β-arrestin to PTH_1_R contributes to desensitization (Feinstein et al., 2011), but it also recruits components of additional signalling pathways (Gesty-Palmer et al., 2006) and initiates internalization of active PTH_1_R–Gs–adenylyl cyclase signalling complexes through β-arrestin- and dynamin-dependent endocytosis (Ferrandon et al., 2009; Gidon et al., 2014). These complexes then continue to generate cAMP from early endosomal compartments (Feinstein et al., 2011; Ferrandon et al., 2009; Wehbi et al., 2013). Similar agonist-evoked internalization of functional signalling pathways occurs for some other GPCRs (Calebiro et al., 2010; Irannejad et al., 2013). The significance for the present work is that internalized PTH_1_R signalling complexes and those at the plasma membrane might deliver cAMP to different intracellular compartments.

The links between the cAMP and Ca^2+^ signals evoked by PTH_1_R are complex (Taylor and Tovey, 2012). In most, although not all, cells (Mahon, 2012), PTH_1_R activates Gs, stimulation of adenylyl cyclase and so formation of cAMP. When PTH_1_R or Gq is expressed at high levels, PTH_1_R can also stimulate phospholipase C (PLC) (Taylor and Tovey, 2012), which catalyses formation of inositol 1,4,5-trisphosphate (IP_3_), and hence Ca^2+^ release from intracellular stores. Typically, such Ca^2+^ signals are evoked by higher concentrations of PTH than are required for stimulation of adenylyl cyclase (Cupp et al., 2013; Okazaki et al., 2008; Takasu et al., 1999; Taylor and Tovey, 2012; van der Lee et al., 2013). Furthermore, some analogues of PTH favour coupling of PTH_1_R to adenylyl cyclase through Gs, whereas others favour PLC coupling (Cupp et al., 2013; Fujimori et al., 1991; Gesty-Palmer and Luttrell, 2011; Takasu et al., 1999) (supplementary material Table S1). Association of PTH_1_R with the scaffold proteins, Na^+^/H^+^ exchange regulatory factors-1 and 2 (NHERF-1 and 2), both of which are expressed in HEK cells (Wang et al., 2010), favours coupling, via Gq or Gi/o, to PLCβ (Wang et al., 2007). Cyclic AMP can also stimulate IP_3_ formation because binding of cAMP to exchange protein-activated by cAMP 1 (EPAC1, also known as RAPGEF3) allows it to activate the small G protein Rap2B, which then stimulates PLCε (Schmidt et al., 2001).

**Fig. 1.**
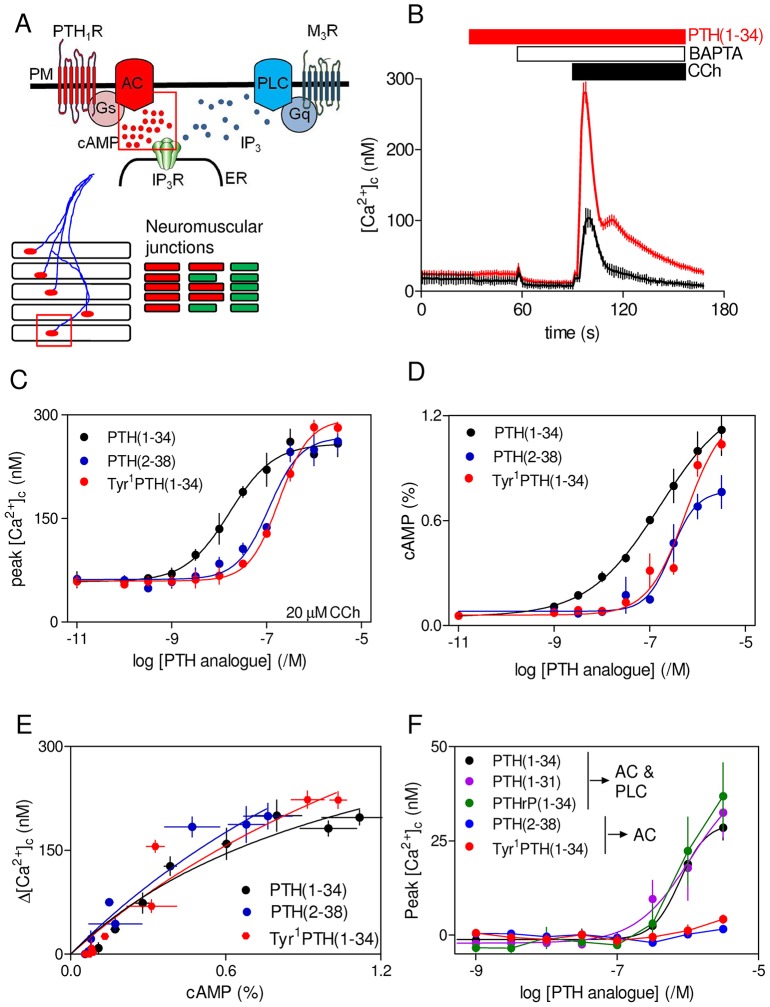
**Potentiation of carbachol-evoked Ca^2+^ signals by PTH(1-34) is mediated by cAMP.** (A) Local delivery of cAMP to IP_3_R within ‘signalling junctions’ (red box) allows stimulation of PTH_1_R to increase the sensitivity of IP_3_Rs to IP_3_. This potentiates the Ca^2+^ release evoked by IP_3_ produced in response to activation of M_3_ muscarinic acetylcholine receptors (M_3_R). All-or-nothing activation of these signalling junctions is analogous to the behaviour of focally innervated skeletal muscle (lower panel), where release of acetylcholine at the neuromuscular junction (red box) evokes all-or-nothing contraction of individual myofibrils. Graded contraction of the muscle fibre then results from recruitment of contracting myofibrils (right panels). See text for further explanation. AC, adenylyl cyclase; PM, plasma membrane; ER, endoplasmic reticulum. (B) Typical changes in [Ca^2+^]_c_ from a population of HEK-PR1 cells stimulated with a submaximal concentration of carbachol (CCh, 20 µM) alone (black trace) or with PTH(1-34) (100 nM, added 1 min before carbachol, red trace). BAPTA (2.5 mM) was added before carbachol to chelate extracellular Ca^2+^. Results are mean±s.d. from two wells in a single experiment. (C) Summary results show concentration-dependent effects of PTH analogues added 1 min before 20 µM carbachol. (D) Effects of PTH analogues on intracellular cAMP measured after 1 min under conditions identical to those used for measurements of [Ca^2+^]_c_. Results show [^3^H]cAMP as a percentage of [^3^H]ATP, [^3^H]ADP and [^3^H]cAMP. Results in C and D are mean±s.e.m. from at least three experiments. (E) Results from C and D were used to establish the relationship (mean±s.e.m.) between cAMP and the potentiated carbachol-evoked increases in [Ca^2+^]_c_ for cells stimulated with the indicated PTH analogues for 1 min. (F) Concentration-dependent effects of PTH analogues alone on the peak increases in [Ca^2+^]_c_ (mean±s.e.m., *n*≥3). The abilities of the analogues to stimulate PLC and/or adenylyl cyclase are shown.

We have shown that in HEK cells stably expressing human PTH_1_R (HEK-PR1 cells), PTH(1-34) stimulates adenylyl cyclase. The cAMP produced directly sensitizes IP_3_ receptors (IP_3_Rs) to the IP_3_ produced when receptors, like endogenous M_3_ muscarinic receptors, stimulate PLC. Hence, concentrations of PTH(1-34) that do not alone evoke increases in [Ca^2+^]_c_ potentiate the Ca^2+^ signals evoked by carbachol, which activates muscarinic receptors (Short and Taylor, 2000; Tovey et al., 2008; Tovey et al., 2003; Tovey and Taylor, 2013). This potentiation is mediated by cAMP, but it requires the cAMP to be delivered at high concentrations from adenylyl cyclase to IP_3_R within a signalling complex that includes AC6 and IP_3_R2. Furthermore, from evidence that even substantial inhibition of adenylyl cyclase failed to attenuate signalling from PTH_1_R to IP_3_R, we proposed that within each signalling complex, cAMP is presented at concentrations more than sufficient to maximally sensitize associated IP_3_Rs (Tovey et al., 2008). We describe the adenylyl cyclase–IP_3_R complex as a ‘signalling junction’ to capture an analogy with the neuromuscular junction of focally innervated skeletal muscle (Fig. 1A), where release of acetylcholine from presynaptic terminals saturates postsynaptic receptors and leads to all-or-nothing contraction of the myofibril. Graded contractions of the muscle then result from graded recruitment of these all-or-nothing fibrillar responses. Because this mode of signalling to IP_3_R requires its close association with adenylyl cyclase, we assessed whether the association is maintained during sustained stimulation with PTH(1-34) when PTH_1_R signalling pathways might be reconfigured. We show that sustained stimulation with PTH leads to diminished potentiation of carbachol-evoked Ca^2+^ signals. This does not require internalization of PTH_1_R. We provide evidence that the hyperactive cAMP signalling junctions that mediate sensitization of IP_3_R by PTH also cause inhibition of IP_3_ formation during sustained stimulation. Our results suggest that delivery of cAMP to its targets within signalling junctions allows rapid potentiation of IP_3_R activity followed by a more slowly developing inhibition of IP_3_ accumulation.

## RESULTS

### PTH potentiates carbachol-evoked Ca^2+^ release through cAMP-mediated sensitization of IP_3_Rs

In HEK-PR1 cells, concentrations of PTH(1-34) that did not alone stimulate Ca^2+^ release potentiated the Ca^2+^ signals evoked by carbachol (Fig. 1B) (Tovey et al., 2008). The effects of PTH(1-34), added 1 min before addition of a submaximal concentration of carbachol (20 µM), were dependent on the concentration of PTH(1-34) (Fig. 1C). Similar results, and with similar sensitivity to PTH(1-34), were obtained using a maximally effective concentration of carbachol (supplementary material Table S2).

At the highest concentrations used (>300 nM), PTH(1-34) alone evoked small (<40 nM) increases in [Ca^2+^]_c_ (Short and Taylor, 2000) (Fig. 1F) that were unaffected by inhibition of adenylyl cyclase, cyclic nucleotide phosphodiesterases (PDEs), protein kinase A (PKA) or EPACs (supplementary material Fig. S1, which also illustrates the targets of the inhibitors used). We do not detect stimulation of PLC by PTH(1-34) in HEK-PR1 cells (Short and Taylor, 2000; Tovey et al., 2008; Tovey and Taylor, 2013), but in some settings PTH_1_R can activate Gq and PLC (see Introduction). We have shown previously that an analogue of PTH, PTH(1-31), that stimulates adenylyl cyclase but was thought not to stimulate PLC, mimicked PTH(1-34) by potentiating carbachol-evoked Ca^2+^ signals (Tovey et al., 2008). By contrast, PTH(3-34), which was thought to selectively activate Gq (Fujimori et al., 1991; but see Takasu et al., 1999), was ineffective (Tovey et al., 2008). A recent study challenges the utility of both analogues (Cupp et al., 2013). In CHO cells expressing PTH_1_R, PTH(1-31) was indistinguishable from PTH(1-34) in stimulating adenylyl cyclase and PLC (Takasu et al., 1999); whereas PTH(3-34) stimulated adenylyl cyclase (with very low potency), but not PLC (Cupp et al., 2013). In the same study, PTH(2-38) and Tyr^1^PTH(1-34) were as effective as PTH(1-34) in stimulating adenylyl cyclase, but they failed to activate PLC (Cupp et al., 2013) (supplementary material Table S1). Selective activation of adenylyl cyclase by PTH(2-38) and Tyr^1^PTH(1-34) is consistent with evidence that N-terminal modifications of PTH attenuate coupling to PLC (Cupp et al., 2013; Takasu et al., 1999).

In HEK-PR1 cells, PTH(2-38) and Tyr^1^PTH(1-34) mimicked PTH(1-34) in both stimulating adenylyl cyclase and potentiating carbachol-evoked Ca^2+^ signals (Fig. 1C,D; supplementary material Table S3). Furthermore, the relationship between the change in intracellular cAMP concentration and the potentiated Ca^2+^ signals was indistinguishable for the three analogues (Fig. 1E). However, whereas the highest concentrations of PTH(1-34), PTHrP(1-34) and PTH(1-31) directly evoked small Ca^2+^ signals, there was no direct response to PTH(2-38) or Tyr^1^PTH(1-34) (Fig. 1F). These results demonstrate that only analogues reported to stimulate PLC directly evoke Ca^2+^ signals, and only at much higher concentrations than are required to potentiate carbachol-evoked Ca^2+^ signals. All the PTH analogues that stimulated adenylyl cyclase also potentiated carbachol-evoked Ca^2+^ signals. These results reinforce our conclusion that cAMP mediates the ability of PTH(1-34) to potentiate carbachol-evoked Ca^2+^ signals (Tovey et al., 2008) (Fig. 1A). That conclusion is consistent with the observation that for all effective PTH analogues, potentiation of carbachol-evoked Ca^2+^ signals was invariably evoked by lower concentrations of PTH (higher pEC_50_, where pEC_50_ is the negative log of the half-maximally effective concentration) than was cAMP accumulation (Fig. 1C,D; supplementary material Table S3). The Ca^2+^ signals evoked by very high concentrations of PTH(1-34) probably result from stimulation of PLC. Our inability to detect IP_3_ formation under these conditions (Tovey et al., 2008; Tovey and Taylor, 2013) is unsurprising when the Ca^2+^ signals evoked by PTH are small and they are detected only under conditions when the IP_3_-evoked Ca^2+^ release is also maximally potentiated by the cAMP produced in response to PTH.

### Potentiation of carbachol-evoked Ca^2+^ release by PTH requires neither PKA nor EPACs

We have previously provided evidence that the effects of PTH(1-34) on carbachol-evoked Ca^2+^ signals require neither PKA nor EPACs (Tovey et al., 2008). The latter conclusion came from experiments in which a membrane-permeant analogue of cAMP that selectively activates EPACs (8-Br-2′-*O*-Me-cAMP) did not mimic the effects of PTH(1-34) or 8-Br-cAMP on carbachol-evoked Ca^2+^ signals. That conclusion is strengthened by results with a new membrane-permeant antagonist of EPAC1 and EPAC2 (also known as RAPGEF4) (ESI-09) (Almahariq et al., 2013). ESI-09 (10 µM, 5 min) had no significant effect on the Ca^2+^ signals evoked by carbachol alone, the concentration-dependent potentiation by PTH(1-34) on carbachol-evoked Ca^2+^ signals or the small Ca^2+^ signals directly evoked by high concentrations of PTH(1-34) (supplementary material Fig. S1E; Fig. S2A,B). It was impracticable to use higher concentrations of ESI-09 or more prolonged treatments because they directly inhibited carbachol-evoked Ca^2+^ release (supplementary material Fig. S2A,C). Others have also recently reported non-specific effects of ESI-09 (Rehmann, 2013). A competitive antagonist of EPACs like ESI-09 might be ineffective if high concentrations of cAMP are locally delivered to IP_3_Rs from adenylyl cyclase (Tovey et al., 2008). However, potentiation of carbachol-evoked Ca^2+^ signals by 8-Br-cAMP, which is uniformly distributed in the cytosol, was also unaffected by ESI-09 (supplementary material Fig. S2D).

These results confirm that EPACs and PKA are not involved in the potentiation of carbachol-evoked Ca^2+^ signals by PTH(1-34) or the direct effects of high concentrations of PTH(1-34) on Ca^2+^ signals. The latter, with evidence that some analogues of PTH stimulate adenylyl cyclase without directly evoking Ca^2+^ signals (Fig. 1D,F), suggests that </emph>EPAC-mediated activation of PLCε (Schmidt et al., 2001) does not contribute to PTH-evoked Ca^2+^ signals in HEK-PR1 cells. We conclude, and consistent with previous work (Tovey et al., 2008), that in HEK-PR1 cells the effects of PTH(1-34) on carbachol-evoked Ca^2+^ release are mediated by cAMP, which sensitizes IP_3_Rs to IP_3_ without need for activation of PKA or EPACs (Fig. 1A).

### Sustained stimulation with PTH reduces potentiation of carbachol-evoked Ca^2+^ signals

PTH(1-34) stimulates delivery of cAMP to IP_3_Rs within signalling junctions (Tovey et al., 2008; Tovey and Taylor, 2013). This, together with evidence that stimulation of adenylyl cyclase at the plasma membrane is followed by internalization of functional PTH_1_R–adenylyl cyclase signalling complexes (see Introduction), prompted us to examine responses of HEK-PR1 cells to carbachol after sustained stimulation with PTH(1-34).

**Fig. 2.**
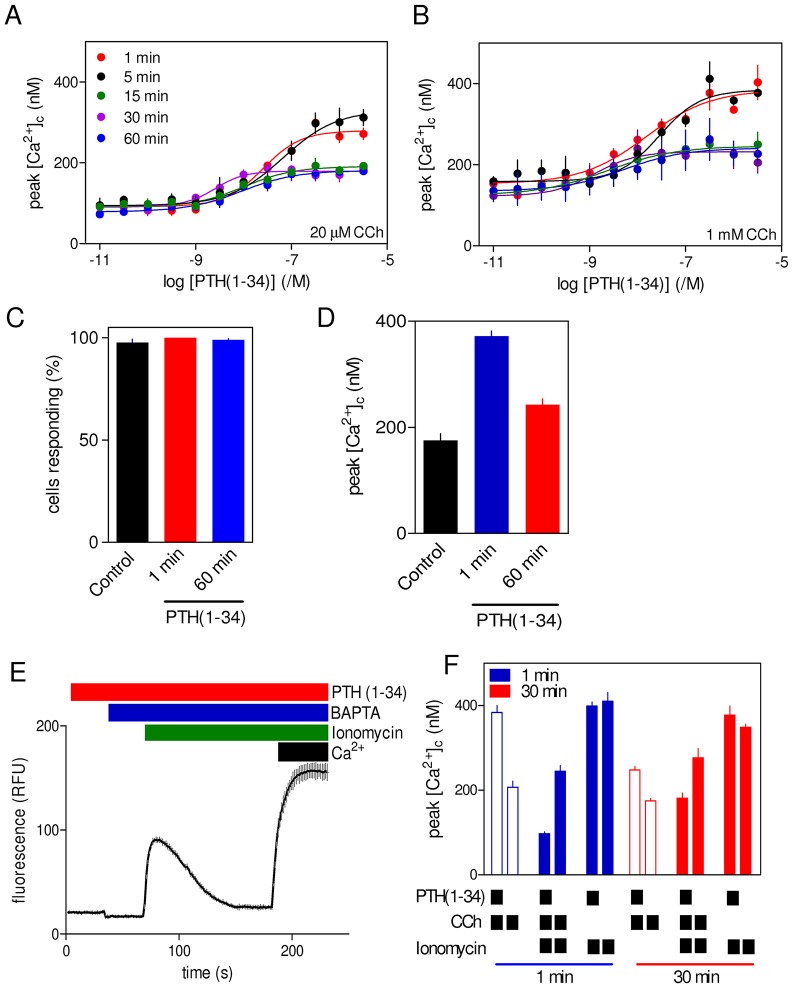
**Sustained stimulation with PTH(1-34) reduces potentiated carbachol-evoked Ca^2+^ signals without affecting the Ca^2+^ content of the intracellular stores.** (A,B) Populations of HEK-PR1 cells were incubated with the indicated concentrations of PTH(1-34) for 1–60 min in HBS before addition of BAPTA (2.5 mM) and either 20 µM (A) or 1 mM carbachol (CCh) (B). The code in A applies to both panels. Results in A and B are mean±s.e.m., *n* = 4. (C,D) Single-cell analyses show the percentage of cells in which carbachol (1 mM) evoked a detectable increase in [Ca^2+^]_c_ in control cells or after stimulation with PTH(1-34) (100 nM) for 1 or 60 min (C), and the increase in [Ca^2+^]_c_ evoked by carbachol under each condition (D). In these experiments, normal HBS was replaced by nominally Ca^2+^-free HBS 5 min before addition of carbachol. Results in C and D are from three coverslips each with ∼65 cells and are presented as mean±s.e.m. (E) The effect of prolonged stimulation with PTH(1-34) on the Ca^2+^ contents of the intracellular stores was assessed by incubating populations of cells with PTH(1-34) for 30 min, before addition of BAPTA (2.5 mM) and then ionomycin (1 µM). Restoration of extracellular Ca^2+^ (10 mM) at the end of the experiment confirmed that the indicator was not saturated by the Ca^2+^ signals evoked by ionomycin. Results show a typical trace from five wells in one experiment. RFU, relative fluorescence units. (F) Similar experiments to that shown in E showing the effects of treatment for 1 or 30 min with PTH(1-34) (100 nM) on the peak Ca^2+^ signals evoked in Ca^2+^-free HBS by carbachol (20 µM, unfilled bars) or ionomycin (1 µM, solid blue and red bars) with or without prior carbachol treatment. Results are mean±s.e.m., *n* = 3.

Varying the duration of the incubation with PTH(1-34) before addition of carbachol established that sustained exposure to PTH(1-34) reduced the maximal amplitude of the carbachol-evoked Ca^2+^ signals by ∼50%, while increasing the sensitivity to PTH(1-34) by almost 10-fold (Fig. 2A,B; supplementary material Table S2). These effects were apparent after 15–30 min, and were not further increased by extending the incubation with PTH(1-34) to 60 min. The inhibition (∼50%) was similar whether maximal or submaximal carbachol concentrations were used to evoke the Ca^2+^ signals. Analyses of single cells showed that the reduced maximal response after prolonged incubation with PTH(1-34) was due to diminished Ca^2+^ signals within individual cells rather than to fewer cells responding (Fig. 2C,D). The diminished amplitude of the potentiated Ca^2+^ signals was not due to loss of Ca^2+^ from intracellular stores. Neither Tyr^1^PTH(1-34) nor PTH(2-38) directly stimulated Ca^2+^ release from intracellular stores (Fig. 1F), but responses to carbachol after brief and sustained stimulation with these analogues were similar to those evoked by equivalent treatments with PTH(1-34) (supplementary material Table S3). Furthermore, addition of ionomycin to cells in Ca^2+^-free HEPES-buffered saline (HBS) to assess the Ca^2+^ contents of the stores after incubation with PTH(1-34) showed that the increase in [Ca^2+^]_c_ evoked by ionomycin was unaffected by acute or sustained stimulation with PTH(1-34) (Fig. 2E,F). The indistinguishable responses were not due to saturation of the Ca^2+^ indicator because restoration of extracellular Ca^2+^ after ionomycin evoked a much larger increase in fluo 4 fluorescence (Fig. 2E). Using similar methods to measure the residual Ca^2+^ content of the stores after stimulation with PTH(1-34) and carbachol, showed that more Ca^2+^ remained within the stores of cells stimulated with carbachol after prolonged treatment with PTH(1-34) (the peak increase in [Ca^2+^]_c_ was 181±12 nM, mean±s.e.m.) than after brief treatment (97±4 nM, *P*<0.05) (Fig. 2F). This again indicates that diminished responses after sustained treatment with PTH(1-34) are not due to loss of Ca^2+^ from intracellular stores. We conclude that sustained stimulation with PTH(1-34) reduces the maximal potentiation of carbachol-evoked Ca^2+^ signals without affecting the Ca^2+^ content of the stores.

### Sustained stimulation with PTH reduces intracellular concentrations of IP_3_

**Fig. 3.**
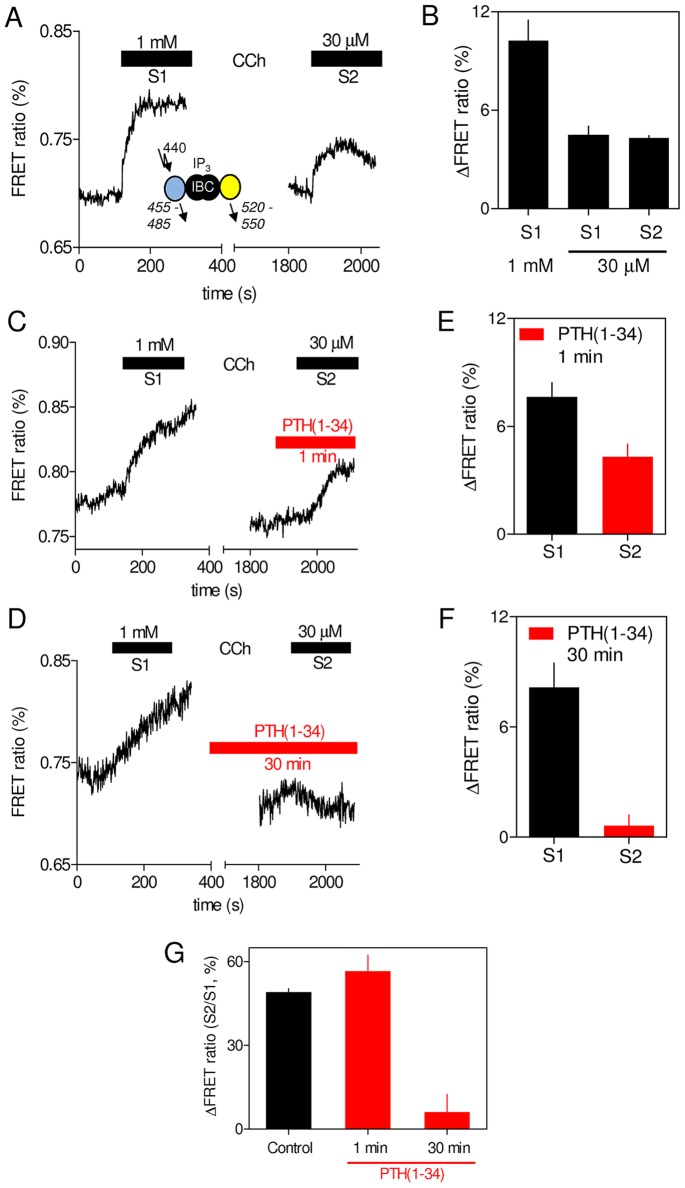
**Sustained stimulation with PTH(1-34) reduces carbachol-evoked increases in cytosolic IP_3_ concentration.** (A) Cytosolic IP_3_ was measured in single HEK-PR1 cells using a FRET sensor during stimulation (3 min) with 1 mM carbachol (CCh) (S1) and then, after washing, with 30 µM carbachol added 30 min later (S2). The trace shows typical results from a cell with no intervening PTH treatment. FRET is denoted as CFP:YFP fluorescence ratio, so that an increased signal (decreased FRET) corresponds to an increase in IP_3_ concentration (see Materials and Methods). The inset shows the IP_3_ sensor with excitation and emission (italics) wavelengths in nm. IBC, IP_3_-binding core. (B) Summary results (mean±s.e.m. for 62 cells from six coverslips) showing ΔFRET (stimulated/basal signal) for cells stimulated with the indicated carbachol concentrations presented as either the first (S1) or second stimulus (S2, i.e. after 1 mM carbachol). (C,D) Typical results from single cells subject to similar treatments to those in A, but with PTH(1-34) (100 nM) added 1 min (C) or 30 min (D) before, and then during, the second addition of carbachol. (E,F) Summary results show ΔFRET for the first and second carbachol stimulation (S1 and S2) as mean±s.e.m. for 36 and 34 cells from five (E) and seven (F) coverslips. (G) For each cell, ΔFRET measurements for the first (S1, 1 mM carbachol) and second stimulus (S2, 30 µM carbachol) were used to calculate S2/S1 for the indicated treatments. Results are mean±s.e.m. for 28–36 cells.

The effects of acute and sustained stimulation with PTH(1-34) on the changes in cytosolic IP_3_ concentration evoked by a submaximal concentration of carbachol (30 µM) were measured in single HEK-PR1 cells using a Förster resonance energy transfer (FRET)-based IP_3_ sensor. Cells were first stimulated with 1 mM carbachol (3 min, S1) to identify responsive cells (Fig. 3A). After washing and a 30-min recovery interval, cells were then stimulated with 30 µM carbachol (3 min, S2). The dual-stimulation protocol, with PTH(1-34) (100 nM) added 1 min or 30 min before the second carbachol stimulus, allowed paired single-cell comparisons of treatments (S2∶S1). This analysis reduced the variability arising from the limited dynamic range of the sensor. The control response shows that the FRET signal evoked by 30 µM carbachol was less than that with 1 mM carbachol (Fig. 3A), and it was unaffected by prior exposure to 1 mM carbachol (Fig. 3B). These results confirm that the sensor was not saturated by the experimental stimulus. Addition of PTH(1-34) 1 min before the second challenge had no effect on the response to carbachol (Fig. 3C,E). This is consistent with evidence that acute stimulation with PTH(1-34) does not stimulate PLC in HEK-PR1 cells (Tovey et al., 2008; Tovey and Taylor, 2013). However, a 30-min pretreatment with PTH(1-34) significantly reduced the increase in cytosolic IP_3_ evoked by carbachol (Fig. 3D,F,G). We conclude that sustained stimulation with PTH(1-34) reduces the stimulatory effect of carbachol on the cytosolic levels of IP_3_.

### Internalization of adenylyl cyclase signalling pathways does not mediate sustained effects of PTH on carbachol-evoked Ca^2+^ signals

**Fig. 4.**
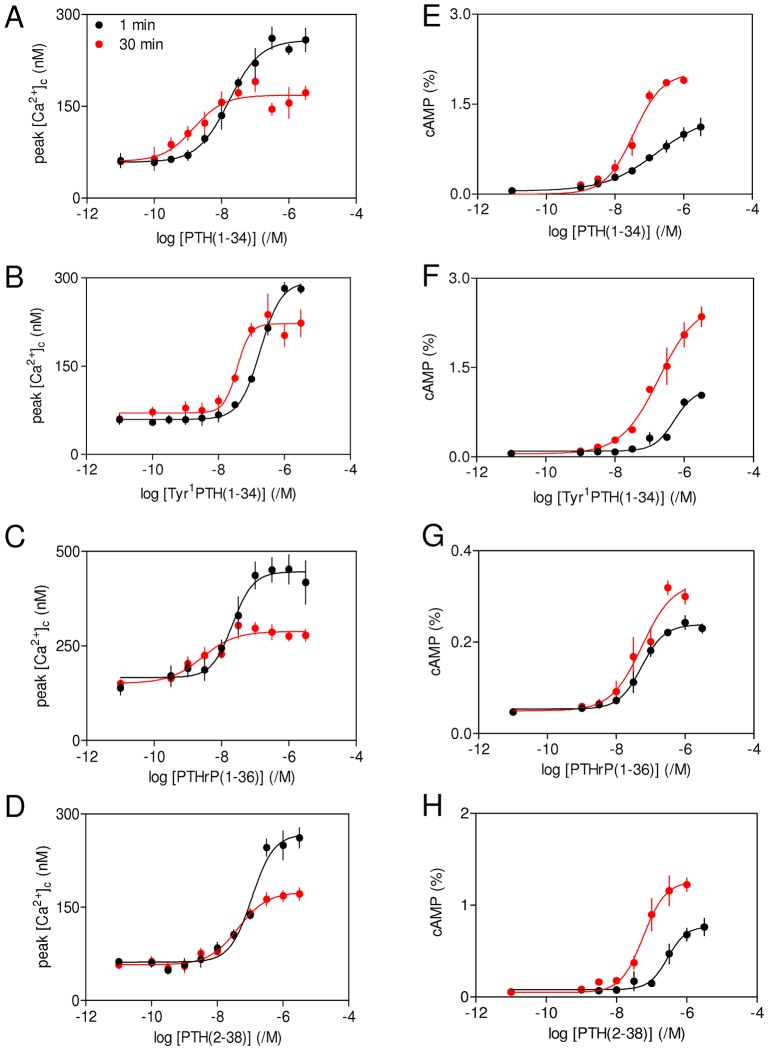
**Internalization of adenylyl cyclase signalling pathways does not contribute to diminished potentiation of Ca^2+^ signals after sustained stimulation with PTH.** (A–D) Cells were stimulated for 1 or 30 min with PTH analogues before addition of carbachol (20 µM) in Ca^2+^-free HBS. The peak increases in [Ca^2+^]_c_ evoked by carbachol are shown. (E–H) Parallel measurements of intracellular cAMP measured under identical conditions. Results are mean±s.e.m. from at least three experiments. The code shown in panel A applies to all panels.

We used PTH analogues that differ in their abilities to evoke internalization of PTH_1_R to assess whether endocytosis of functional adenylyl cyclase signalling pathways contributes to the sustained effects of PTH(1-34) on carbachol-evoked Ca^2+^ signals. PTH(1-34) evokes receptor internalization and sustained signalling from endosomal adenylyl cyclase, PTH(2-38) does not evoke receptor internalization, Tyr^1^PTH(1-34) is a weak partial agonist for receptor internalization and PTHrP(1-36) evokes receptor internalization but no persistent adenylyl cyclase signalling (see supplementary material Table S1). The acute and sustained effects of each analogue on carbachol-evoked Ca^2+^ signals were similar to those evoked by PTH(1-34) (Fig. 4A–D; supplementary material Table S3). For each PTH analogue, the maximal amplitude of the Ca^2+^ signal evoked by carbachol was smaller after sustained stimulation, despite each causing intracellular levels of cAMP to be greater after stimulation for 30 min relative to 1 min (Fig. 4E–H; supplementary material Table S3). Although PTHrP(1-36) mimicked the effects of PTH(1-34) in potentiating carbachol-evoked Ca^2+^ signals, it stimulated less cAMP accumulation. This is unexpected because others have suggested that PTHrP(1-36) (Dean et al., 2008), PTHrP(1-34) and PTHrP(1-37) (Cupp et al., 2013) are as efficacious as PTH(1-34) in stimulating accumulation of cAMP, albeit in cells with 10-fold greater levels of PTH_1_R expression (Dean et al., 2008). We have not further explored this issue. For most PTH analogues, the sensitivity to PTH of both cAMP accumulation and Ca^2+^ signalling increased during sustained stimulation (ΔpEC_50_ values in supplementary material Table S3). This suggests that a component of the increased sensitivity of the Ca^2+^ signals is probably due to the increased sensitivity of adenylyl cyclase activation to PTH during prolonged stimulation. The more important point for the present work is that, for all the PTH analogues, sustained stimulation causes greater accumulation of cAMP, but lesser potentiation of carbachol-evoked Ca^2+^ signals. Collectively, these results suggest that internalization of functional PTH_1_R signalling complexes is unlikely to be responsible for the sustained effects of PTH on carbachol-evoked Ca^2+^ signals. We therefore assessed the effects of more directly evoking sustained elevations in intracellular cAMP concentration on carbachol-evoked Ca^2+^ signals.

**Fig. 5.**
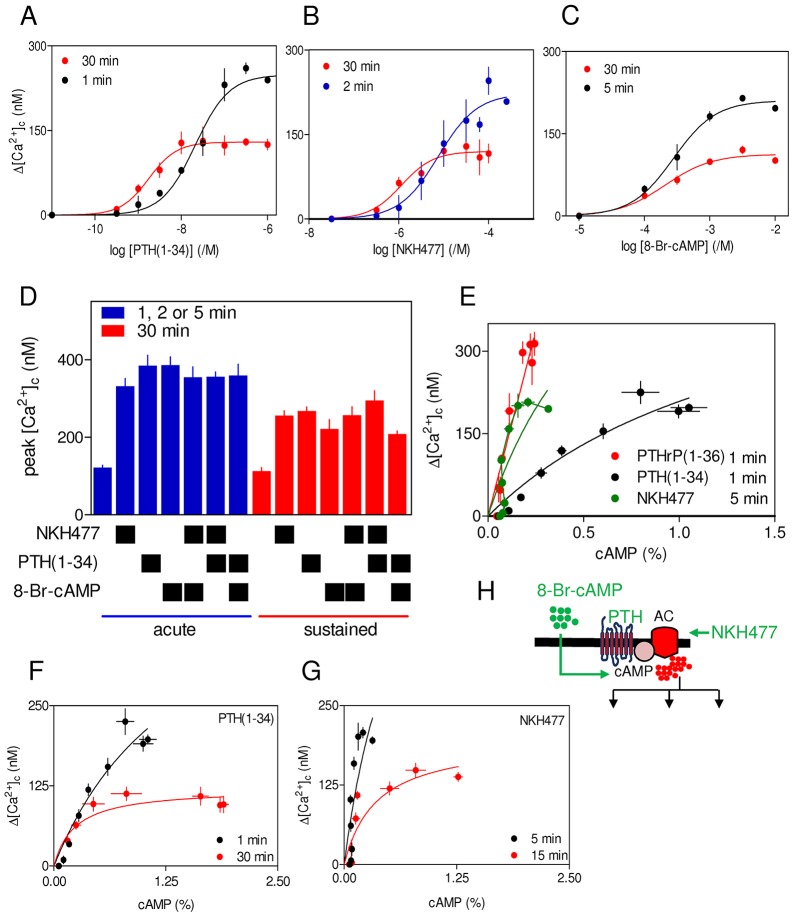
**Diminished potentiation of carbachol-evoked Ca^2+^ signals after sustained increases in intracellular cAMP concentration.** (A–C) Peak increases in [Ca^2+^]_c_ evoked by addition of carbachol (20 µM) in Ca^2+^-free HBS to cells preincubated with PTH(1-34) (A), NKH477 (B) or 8-Br-cAMP (C) for the indicated times. Δ[Ca^2+^]_c_ denotes the difference in the peak increase in [Ca^2+^]_c_ evoked by carbachol alone and after each pretreatment. (D) Similar experiments to those in A–C, show the effects of carbachol (20 µM) on the peak increase in [Ca^2+^]_c_ after the indicated combinations of treatments for 1 min [PTH(1-34)], 2 min (NKH477) or 5 min (8-Br-cAMP) (denoted acute) and 30 min (denoted sustained). (E) Comparison of the relationship between cAMP and Δ[Ca^2+^]_c_ for cells acutely stimulated with PTH(1-34) (1 min, *n* = 7), PTHrP(1-36) (1 min, *n* = 3) or NKH477 (5 min, *n* = 8). (F,G) Relationships between cAMP and Δ[Ca^2+^]_c_ for cells stimulated with carbachol (20 µM) after acute or sustained stimulation with PTH(1-34) (F) or NKH477 (G). Results are means±s.e.m. for *n* = 4 (A–D) or at least *n* = 3 (F,G). (H) Targets of the drugs used. AC, adenylyl cyclase.

Brief stimulation (1–5 min) with 8-Br-cAMP, PTH(1-34) or NKH477, a soluble analogue of forskolin that directly activates adenylyl cyclase (Ito et al., 1993), caused similar potentiation of carbachol-evoked Ca^2+^ signals (Fig. 5A–C) and their maximal effects were non-additive (Fig. 5D). Because the three stimuli take different times to reach their targets, incubation periods (1–5 min) were optimized for each to achieve maximal potentiation of carbachol-evoked Ca^2+^ signals. The results extend previous work (Tovey et al., 2008) by confirming that cAMP alone mediates potentiation of carbachol-evoked Ca^2+^ signals by PTH(1-34). However, the relationship between intracellular cAMP and Δ[Ca^2+^]_c_ is different for PTH(1-34), PTHrP(1-36) and NKH477 (Fig. 5E): the effects of PTH(1-34) on Ca^2+^ signals are associated with much larger accumulations of cAMP than are comparably potentiated Ca^2+^ signals evoked by PTHrP(1-36) or NKH477. This indicates that IP_3_R cannot be responding to a uniformly delivered global increase in cytosolic cAMP.

**Fig. 6.**
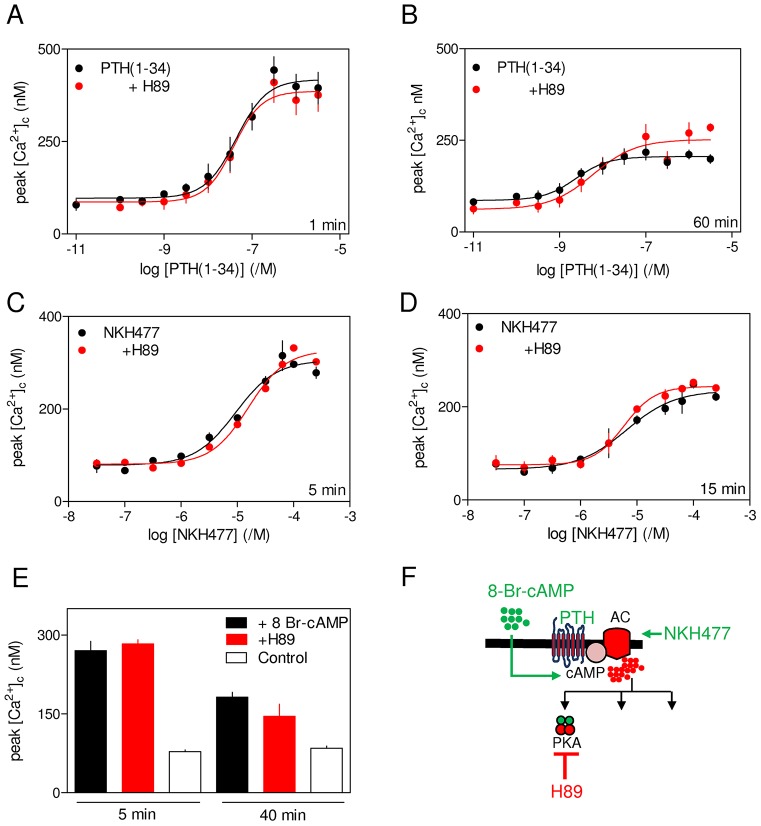
**Neither acute nor sustained potentiation of carbachol-evoked Ca^2+^ signals requires activation of PKA.** (A–E) Cells were incubated with H89 (10 µM, 20 min) to inhibit PKA before acute or sustained stimulation with PTH(1-34) (A,B), NKH477 (C,D) or 8-Br-cAMP (10 mM) (E) followed by addition of carbachol (20 µM) in Ca^2+^-free HBS. Results show peak increases in [Ca^2+^]_c_ evoked by carbachol as mean±s.e.m., *n* = 3. (F) Targets of the drugs used. AC, adenylyl cyclase.

Sustained exposure to PTH(1-34), NKH477 or 8-Br-cAMP caused similar decreases in the maximal potentiation of carbachol-evoked Ca^2+^ signals, and again the maximal effects of each were non-additive (Fig. 5A–D; supplementary material Tables S4 and S5). For both PTH(1-34) and NKH477, prolonged stimulation reduced the apparent effectiveness of cAMP in potentiating carbachol-evoked Ca^2+^ signals (Fig. 5F,G). Neither the acute nor sustained effects of PTH(1-34), NKH477 or 8-Br-cAMP were affected by inhibition of PKA, because treatment with the PKA inhibitor H89 had no effect (Tovey et al., 2008) (Fig. 6; supplementary material Fig. S3A). There was also no effect of H89 on the amount of cAMP produced after stimulation with PTH(1-34) for 1 min or 60 min. For cells treated with H89, amounts of intracellular cAMP detected after stimulation with 3 µM PTH(1-34) for 1 min or 60 min were 95±0.1% and 98±0.2% (mean±s.e.m.) of those detected in matched control cells (*n* = 3) (supplementary material Fig. S3B). We conclude that sustained elevations of intracellular cAMP, whether evoked by activation of PTH_1_R or directly, attenuate the potentiation of carbachol-evoked Ca^2+^ signals. Neither the potentiation of Ca^2+^ signals by cAMP nor the diminished response after sustained elevation of cAMP requires activation of PKA.

### Brief and sustained stimulation with PTH potentiate carbachol-evoked Ca^2+^ signals through cAMP junctions

Although cAMP mediates the effects of PTH on carbachol-evoked Ca^2+^ signals (Tovey et al., 2008), sustained exposure to PTH causes a more substantial increase in intracellular cAMP than acute stimulation, but a lesser potentiation of carbachol-evoked Ca^2+^ signals (Fig. 4; supplementary material Table S3). The reduced effectiveness of cAMP with increased duration of exposure is clear from comparison of the relationships between cAMP and potentiated Ca^2+^ signals for cells stimulated acutely or chronically with PTH(1-34) or NKH477 (Fig. 5F,G).

**Fig. 7.**
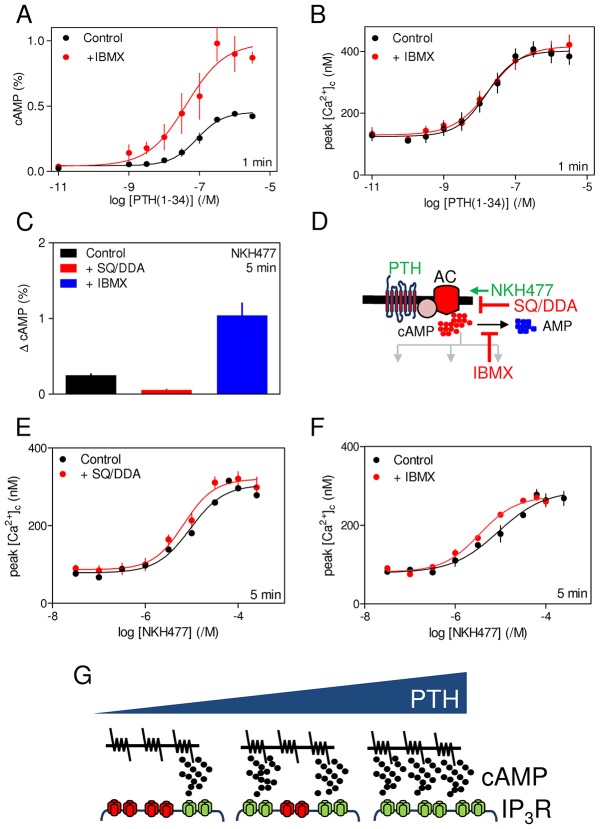
**Acute potentiation of carbachol-evoked Ca^2+^ signals through cAMP signalling junctions.** (A,B) Cells were incubated with IBMX (1 mM, 5 min) before stimulation with PTH(1-34) for 1 min and then addition of carbachol (20 µM) in Ca^2+^-free HBS. Results show intracellular levels of cAMP (A) and the peak increases in [Ca^2+^]_c_ evoked by carbachol (B). (C) Effects of IBMX (1 mM, 5 min) or SQ/DDA (1 mM SQ 22536 and 200 µM DDA, 20 min) on the increase in intracellular cAMP concentration evoked by NKH477 (300 µM, 5 min). (D) Targets of the drugs used. AC, adenylyl cyclase. (E,F) Effects of similar treatments with IBMX of SQ/DDA on the peak Ca^2+^ signals evoked by carbachol (20 µM) after incubation with the indicated concentrations of NKH477 for 5 min. Results in A–C,E,F are mean±s.e.m., *n* = 3. (G) Communication between PTH_1_R and IP_3_Rs is proposed to be mediated by local delivery of supramaximal concentrations of cAMP from adenylyl cyclase to IP_3_Rs within junctional complexes. We suggest that the concentration-dependent effects of PTH are then mediated by recruitment of these all-or-nothing junctions, rather than from graded activity within each (Tovey et al., 2008).

Acute (1 min) potentiation of carbachol-evoked Ca^2+^ signals by PTH(1-34) was unaffected by substantially inhibiting cAMP formation (by inhibiting adenylyl cyclase with SQ22536 and DDA, hereafter denoted SQ/DDA) or degradation (by inhibiting cyclic nucleotide PDEs with IBMX), although both treatments had the expected effects on intracellular cAMP (Fig. 7A,B; supplementary material Table S6; Fig. S3C,D). Supplementary material Fig. S3E demonstrates that if the cAMP that regulates IP_3_R were uniformly distributed, the observed 60–70% inhibition of adenylyl cyclase by SQ/DDA would cause a detectable inhibition of the effects of PTH(1-34) on carbachol-evoked Ca^2+^ signals. The lack of effect of SQ/DDA on Ca^2+^ responses is not, therefore, a limitation of our methods. Similar results were obtained when NKH477 was used to acutely stimulate adenylyl cyclase. SQ/DDA and IBMX had the expected effects on intracellular concentrations of cAMP (Fig. 7C,D), but they had no effect on the potentiation of carbachol-evoked Ca^2+^ signals (Fig. 7E,F). These results confirm previous work, where we argued that the inability of SQ/DDA or IBMX to affect potentiation of carbachol-evoked Ca^2+^ signals by any concentration of acutely presented PTH(1-34), despite substantial effects on intracellular concentrations of cAMP, suggests that cAMP is locally delivered at super-saturating concentrations to IP_3_Rs (Tovey et al., 2008; Tovey and Taylor, 2013). We propose that the concentration-dependent effects of PTH(1-34) then arise from recruitment of these signalling junctions, rather than from graded activity within each (Fig. 7G).

**Fig. 8.**
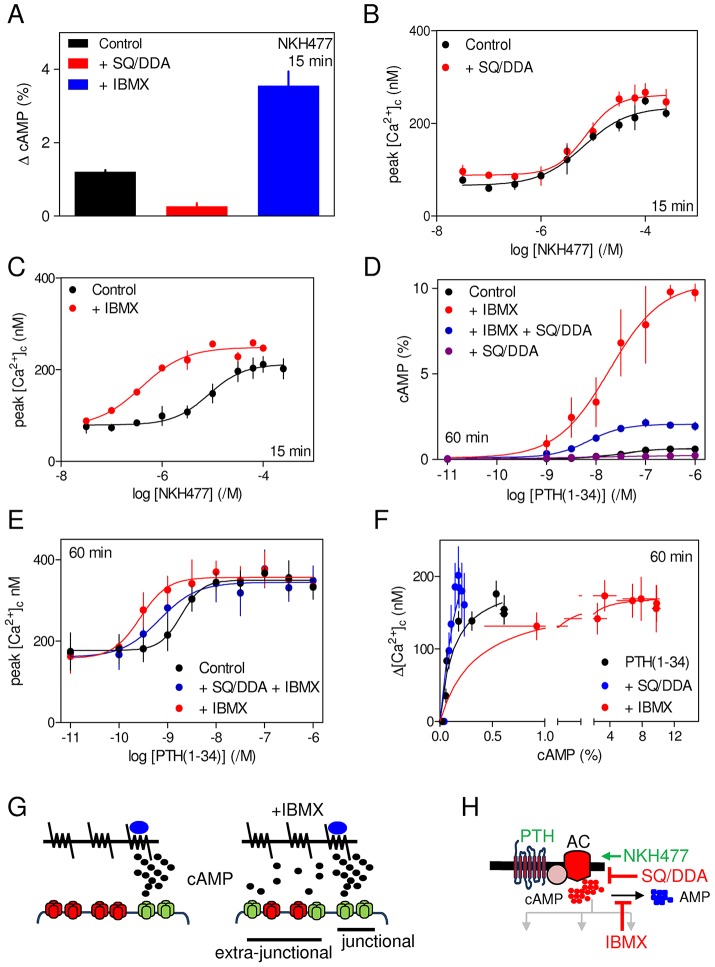
**Sustained potentiation of carbachol-evoked Ca^2+^ signals is mediated by cAMP junctions.** (A) Effects of SQ/DDA and IBMX (concentrations as in Fig. 7C) on the increase in intracellular cAMP concentration evoked by NKH477 (300 µM, 15 min). (B,C) Effects of the same treatments on the peak Ca^2+^ signals evoked by carbachol (20 µM) after incubation for 15 min with the indicated concentrations of NKH477. (D,E) Similar analyses of the effects of SQ/DDA and/or IBMX on the increase in intracellular cAMP concentration evoked by incubation with the indicated concentrations of PTH(1-34) for 60 min (D) or the peak Ca^2+^ signals evoked by carbachol (20 µM) added 60 min after PTH(1-34) (E). Results in A–E are mean±s.e.m., *n* = 3. (F) Relationships between cAMP and Δ[Ca^2+^]_c_ for cells stimulated with PTH(1-34) for 60 min alone or after treatment with SQ/DDA or IBMX (mean±s.e.m., *n* = 3). (G) Normally cAMP is delivered to IP_3_R within signalling junctions (left panel), but the massive accumulation of cAMP during sustained stimulation with PTH and IBMX (right panel) achieves global cytosolic cAMP concentrations sufficient to sensitize IP_3_Rs beyond active junctions. (H) Targets of the drugs used. AC, adenylyl cyclase.

In cells stimulated with PTH(1-34) for 60 min, cAMP formation was reduced by ∼70% after inhibition of adenylyl cyclase by SQ/DDA, but there was no significant effect on the potentiation of carbachol-evoked Ca^2+^ signals (supplementary material Table S6). Similar effects were observed after sustained stimulation with NKH477: cAMP accumulation was substantially inhibited by SQ/DDA without affecting the concentration-dependent effects of NKH477 on carbachol-evoked Ca^2+^ signals (Fig. 8A,B). These results suggest that the sustained effects of PTH or direct activation of adenylyl cyclase on carbachol-evoked Ca^2+^ signals are, like those evoked by acute stimulation, mediated by hyperactive cAMP junctions.

IBMX massively increased the amount of cAMP produced after sustained stimulation with PTH(1-34) or NKH477. In parallel analyses, IBMX significantly increased the sensitivity of carbachol-evoked Ca^2+^ signals to PTH(1-34) and NKH477 without affecting the maximal amplitude of the increase in [Ca^2+^]_c_ (Fig. 8A,C–F; supplementary material Table S6). The latter remained smaller than the increase observed after acute stimulation, demonstrating that even massive increases in intracellular cAMP concentration cannot surmount the attenuation of potentiated Ca^2+^ signals after sustained stimulation with PTH. As with all other analyses, inhibition of PKA (with H89) had no effect on the potentiation of carbachol-evoked Ca^2+^ signals by PTH(1-34) in the presence of IBMX (supplementary material Fig. S4), re-affirming that PKA is not involved in the potentiation of carbachol-evoked Ca^2+^ signals.

Whereas SQ/DDA had no effect on the acute potentiation of Ca^2+^ signals by PTH(1-34) alone or with IBMX (Fig. 7; supplementary material Fig. S3), it partially reversed the increase in sensitivity to PTH(1-34) during sustained stimulation with PTH(1-34) and IBMX (Fig. 8E). These opposing effects of IBMX and SQ/DDA on the sensitivity of carbachol-evoked Ca^2+^ signals to PTH(1-34) confirm the role of cAMP in mediating the effect. Sustained stimulation (60 min) with PTH(1-34) in the presence of IBMX generated levels of intracellular cAMP that were 22-fold greater than those evoked by acute (1 min) stimulation (supplementary material Table S6). Although SQ/DDA substantially inhibited adenylyl cyclase, the amount of intracellular cAMP in cells stimulated with PTH(1-34) for 60 min with IBMX remained substantially greater than during acute stimulation (Fig. 8F; supplementary material Table S6). These results suggest that when the global intracellular cAMP concentration is massively increased, it achieves levels that can sensitize the Ca^2+^ signals evoked by carbachol without the need for cAMP signalling junctions. Under these conditions, cAMP will sensitize both junctional IP_3_R and extra-junctional IP_3_R. It would be expected that recruitment of the latter would be attenuated by inhibition of adenylyl cyclase, whereas junctional signalling would be unaffected (Fig. 8G).

## DISCUSSION

### Signalling from PTH_1_R to Ca^2+^ signals through adenylyl cyclase–IP_3_R junctions

PTH(1-34) potentiates carbachol-evoked Ca^2+^ release by increasing the sensitivity of IP_3_R (Fig. 1A). The potentiated response is mediated by cAMP, it requires neither PKA nor EPACs, and probably results from cAMP binding directly to IP_3_R or closely associated proteins (Tovey et al., 2010; Tovey et al., 2008). Despite cAMP being the essential link between PTH_1_R and Ca^2+^ signalling, acute responses to all concentrations of PTH(1-34) or to direct stimulation of adenylyl cyclase (with NKH477) were insensitive to inhibition of either adenylyl cyclase (with SQ/DDA) or cyclic nucleotide PDEs (with IBMX), although each inhibitor had the expected effect on global concentrations of intracellular cAMP (Fig. 7; supplementary material Table S6; Fig. S3). This, together with the inconsistent relationship between intracellular cAMP and Ca^2+^ signals for different analogues of PTH and direct stimulation of adenylyl cyclase (Fig. 5E), establish that the responses are not mediated by global cAMP signals uniformly delivered to the cytosol. Instead, we suggest that cAMP is delivered to IP_3_R within signalling junctions at concentrations that are more than sufficient to fully sensitize associated IP_3_Rs. We propose that the concentration-dependent effects of PTH(1-34) then result from recruitment of these digital junctions, rather than from graded activity within individual junctions (Fig. 7G) (Tovey et al., 2008). This evidence that potentiation of carbachol-evoked Ca^2+^ signals by PTH(1-34) requires local communication between adenylyl cyclase and IP_3_Rs motivated our analysis of sustained responses to PTH(1-34) during which functional adenylyl cyclase signalling pathways are internalized (see Introduction).

### Sustained signalling from PTH_1_R through adenylyl cyclase–IP_3_R junctions

Sustained stimulation with PTH(1-34) potentiated carbachol-evoked Ca^2+^ signals, but the maximal amplitude of the response was smaller than with acute stimulation, and the sensitivity to PTH(1-34) was increased (Fig. 2A,B; Fig. 4). The latter might, at least in part, be due to an increase in the sensitivity of cAMP accumulation to PTH(1-34) during sustained stimulation (supplementary material Table S3). The diminished Ca^2+^ responses were not due to fewer cells responding or to loss of Ca^2+^ from intracellular stores (Fig. 2), and they were unaffected by inhibition of PKA (Fig. 6). Acute and sustained Ca^2+^ responses to PTH analogues that differ in whether they evoke internalization of functional adenylyl cyclase signalling complexes were similar to those evoked by PTH(1-34) (Fig. 4). Furthermore, acute and sustained responses to 8-Br-cAMP or direct activation of adenylyl cyclase mimicked the responses evoked by PTH(1-34), and the maximal effects of sustained exposure to each stimulus were non-additive (Fig. 5A–D). Collectively, these results suggest that additional effects of active PTH_1_R, like stimulation of phosphatidylinositol 3-kinase and Akt (Yamamoto et al., 2007), are unlikely to contribute to the sustained effects of PTH on carbachol-evoked Ca^2+^ signals. Instead, we conclude that attenuated potentiation of carbachol-evoked Ca^2+^ signals during sustained exposure to PTH(1-34) is mediated by a sustained increase in cytosolic cAMP that does not require PKA or internalization of PTH_1_R signalling complexes.

The insensitivity of the sustained responses to PTH(1-34) and NKH477 to substantial inhibition of adenylyl cyclase (Fig. 8) suggests that hyperactive cAMP signalling junctions regulate the changes in signalling to IP_3_R that occur during sustained activation of adenylyl cyclase. We conclude, and despite evidence that sustained stimulation with PTH(1-34) evokes internalization of functional adenylyl cyclase signalling complexes (Ferrandon et al., 2009), that PTH_1_R retains its ability to signal through hyperactive adenylyl cyclase–IP_3_R signalling junctions during sustained stimulation. Sustained stimulation with PTH(1-34) in the presence of IBMX caused the global concentration of intracellular cAMP to increase to levels sufficient to sensitize IP_3_R without the usual need for junctional delivery of cAMP. This was evident from the increased sensitivity to PTH(1-34) and NKH477 after sustained stimulation in the presence of IBMX, and its partial reversal by inhibition of adenylyl cyclase with SQ/DDA (Fig. 8). Our demonstration that SQ/DDA can, under these experimental conditions, attenuate the effects of PTH(1-34) on carbachol-evoked Ca^2+^ signals reinforces our conclusion that hyperactive cAMP signalling junctions normally mediate the effects of PTH(1-34). Although the global increase in cAMP increased the sensitivity to PTH(1-34) and NKH477, it had no effect on the maximal response, which remained smaller than that evoked by acute stimulation. This demonstrates that ineffective delivery of cAMP to IP_3_R during sustained stimulation does not cause the diminished potentiation of carbachol-evoked Ca^2+^ signals. Instead, sustained increases in intracellular cAMP reduce the accumulation of cytosolic IP_3_ after carbachol stimulation (Fig. 3). We have not addressed whether this results from decreased production or enhanced degradation of IP_3_. However, the diminished responses to carbachol during sustained stimulation with PTH(1-34) are mediated by cAMP (Figs 5–8), independent of PKA (Fig. 6) and dependent on delivery of cAMP within hyperactive signalling junctions (Fig. 8).

We conclude that PTH(1-34) through PTH_1_R stimulates adenylyl cyclase and locally delivers cAMP at supersaturating concentrations to associated IP_3_Rs, thereby increasing their sensitivity to IP_3_ and so potentiating the Ca^2+^ signals evoked by carbachol (Fig. 8G). This junctional delivery of cAMP is maintained during sustained stimulation with PTH(1-34), but prolonged activity of the junctions leads to an inhibition of IP_3_ accumulation. These cAMP junctions, which behave as ‘on–off’, or digital, switches, allow fast and robust signalling from adenylyl cyclase to its targets. The cAMP then mediates both the initial effects of PTH(1-34) on Ca^2+^ signals and the longer term attenuation of the response without need for activation of PKA.

## MATERIALS AND METHODS

### Materials

*N*-[2-[[3-(4-bromophenyl)-2-propenyl]amino]ethyl]-5-isoquinolinesulfonamide dihydrochloride (H89) and 8-Br-cAMP were from R&D Systems (Minneapolis, MN). 2′,5′-dideoxyadenosine (DDA), *N*,*N*-dimethyl-(3*R*,4a*R*,5*S*,6a*S*,10*S*,10a*R*,10b*S*)-5-(acetyloxy)-3-ethenyldodecahydro-10,10b-dihydroxy-3,4a,7,7,10a-pentamethyl-1-oxo-1*H*-naphtho[2,1-*b*]pyran-6-yl ester β-alanine hydrochloride (NKH477) and 9-(tetrahydro-2-furanyl)-9*H*-purin-6-amine (SQ22536) were from Merck Biosciences (Middlesex, UK). [2,8-^3^H]-adenine was from Perkin Elmer (Waltham, MA). 1,2-*bis*(o-aminophenoxy)ethane-N,N,N′,N′-tetraacetic acid (BAPTA) was from Molekula (Gillingham, UK). Carbamylcholine chloride (carbachol, CCh) and 3-isobutyl-1-methylxanthine (IBMX) were from Sigma-Aldrich (Gillingham, UK). Ionomycin was from Apollo Scientific (Stockport, UK). Cell culture media, G-418, fluo 4AM and fura 2AM were from Life Technologies (Paisley, UK). 3-[5-(tert-butyl)isoxazol-3-yl]-2-[2-(3-chlorophenyl)hydrazono]-3-oxopropanenitril (ESI-09) was from Biolog Life Science Institute (Bremen, Germany). All PTH analogues were human forms and supplied by either Bachem (Bubendorf, Switzerland) or, for PTHrP(1-36), custom-synthesized by Selleckchem (Boston, MA, USA). Sequences of the analogues used are listed in supplementary material Table S1.

### Measurements of [Ca^2+^]_c_

HEK-PR1 cells (∼10^5^ PTH_1_R/cell) were cultured as described previously (Tovey et al., 2008). Measurements of [Ca^2+^]_c_ in cell populations were performed as previously described (Tovey et al., 2008). Briefly, confluent cultures of HEK-PR1 grown in 96-well plates were loaded with fluo 4 by incubation with fluo 4AM (2 µM, 20°C) in HEPES-buffered saline (HBS). HBS had the following composition (in mM): NaCl 135, KCl 5.9, MgCl_2_ 1.2, CaCl_2_ 1.5, HEPES 11.6 and glucose 11.5, pH 7.3. After 1 h, loading medium was replaced with HBS, and after 45 min cells were used at 20°C for measurements of [Ca^2+^]_c_. A fluorescence plate-reader equipped to allow automated fluid additions (FlexStation 3, Molecular Devices, Sunnyvale, CA, USA) was used to record fluorescence at intervals of 1.44 s (excitation at 485 nm; emission at >525 nm) (Tovey et al., 2006). Fluorescence (*F*) was calibrated to [Ca^2+^]_c_ from: [Ca^2+^]_c_ = *K*_D_(*F*−*F*_min_)/(*F*_max_−*F*), where *K*_D_ is the equilibrium dissociation constant of fluo 4 for Ca^2+^ (345 nM); *F*_min_ and *F*_max_ were measured from cells treated with Triton X-100 (0.2%, v/v) in the presence of BAPTA (10 mM) or CaCl_2_ (10 mM).

For single-cell measurements of [Ca^2+^]_c_, near-confluent cultures of HEK-PR1 cells were grown on poly-l-lysine-coated round coverslips (22-mm diameter) and loaded with fura 2 by incubation with fura 2AM (2 µM, 45 min, 20°C) in HBS. The medium was removed and cells were incubated for a further 45 min in HBS at 20°C before single-cell imaging using an Olympus IX71 inverted fluorescence microscope. Cells were alternately excited at 5-s intervals with light (340 nm and 380 nm) from a Xe-arc lamp and monochromator, while collecting emitted light at 510 nm using a Luca EMCCD camera (Andor Technology, Belfast, UK) and MetaFluor software (Molecular Devices, Sunnyvale, CA). Autofluorescence was determined at the end of each experiment by addition of ionomycin (1 µM) and MnCl_2_ (10 mM) and subtracted from measurements before computing fluorescence ratios (*R* = *F*_340_/*F*_380_). These were calibrated to [Ca^2+^]_c_ from:
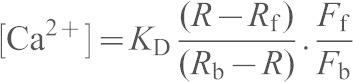
where the *K*_D_ for fura 2 is 224 nM, *R*_b_ and *R*_f_ are the fluorescence ratios for fura 2 with and without Ca^2+^ bound, and *F*_b_ and *F*_f_ are the fluorescence recorded at 380 nM with and without Ca^2+^.

Concentration–effect relationships were fitted to Hill equations using Prism version 5 (GraphPad, San Diego, CA, USA). Results are shown as mean±s.e.m. Statistical comparisons of sensitivities used pEC_50_ values (−log of the half-maximally effective concentration, EC_50_). Because our experiments were performed over a prolonged period using HEK-PR1 cells from different passages, there is some variability in the absolute sensitivities to carbachol and PTH(1-34), and in the amplitudes of the Ca^2+^ signals evoked. All statistical comparisons are therefore between experiments performed in parallel and analysed using paired Student's *t*-tests.

### Measurements of intracellular cAMP

These assays were performed under conditions that replicate those used for measurements of [Ca^2+^]_c_. HEK-PR1 cells were grown in 24-well plates until ∼90% confluent, [^3^H]adenine (2 µCi per well) was then added to the culture medium. After 2 h at 37°C in 5% CO_2_, the medium was removed, cells were washed with HBS, and used for experiments in HBS at 20°C. Because many cells extrude cAMP into the extracellular medium (Copsel et al., 2011), reactions were terminated by first removing the medium and then adding ice-cold trichloroacetic acid (5% v/v, 1 ml). After 30 min on ice, [^3^H]cAMP was separated from other ^3^H-adenine nucleotides by sequential column chromatography on Dowex cation exchange resin and alumina as previously described (Pantazaka et al., 2013). The activity of the eluates was determined by liquid scintillation counting and amounts of [^3^H]cAMP are expressed as percentages of the sum of the activities recovered in the [^3^H]cAMP, [^3^H]ADP and [^3^H]ATP fractions.

### Measurements of intracellular IP_3_

A FRET sensor based on the IP_3_-binding core of IP_3_R1 (Tovey and Taylor, 2013) was used to measure cytosolic concentrations of IP_3_ in single HEK-PR1 cells under conditions similar to those used for measurements of [Ca^2+^]_c_. The plasmid and properties of the sensor were as described previously (Tovey and Taylor, 2013). The sensor comprises the IP_3_-binding core attached through short linkers to enhanced cyan fluorescent protein (CFP) at its N-terminus and enhanced yellow fluorescent protein (YFP) at its C-terminus (see inset to Fig. 3A). IP_3_ binding causes a decrease in FRET. HEK-PR1 cells on poly-l-lysine-coated, 22-mm diameter, glass coverslips were grown for 48 h in 6-well plates to ∼60% confluence. Cells were then transiently transfected with plasmid encoding the IP_3_ sensor (1 µg) using Lipofectamine LTX reagent with PLUS reagent, according to the manufacturer's instructions (Life Technologies, Paisley, UK). Cells were imaged after 48 h. An Olympus IX71 inverted fluorescence microscope with a 40× objective and a 440 nm/520 nm dual band-pass dichroic mirror was used to record fluorescence from widefield images after excitation at 440 nm (to excite CFP). A Luca EMCCD camera (Andor Technology, Belfast, UK) was used to collect emitted fluorescence simultaneously at 1-s intervals from YFP (520–550 nm) and CFP (455–485 nm) using a Cairn Optosplit 2 image-splitter fitted with a 495-nm dichroic mirror. After correction for background fluorescence (determined from cytosolic areas of non-transfected cells), FRET ratios are presented as the ratio of CFP emission to YFP emission, so that the ratio increases (decreased FRET) after IP_3_ binding. The transfection efficiency was ∼65%, and 52±2% (mean±s.e.m., *n* = 17 coverslips) of transfected cells responded to carbachol (1 mM) with discernible FRET changes; only these responsive cells were included in analyses of the effects of PTH(1-34).

## Supplementary Material

Supplementary Material

## References

[b1] Almahariq M. Tsalkova T. Mei F. C. Chen H. Zhou J. Sastry S. K. Schwede F. Cheng X. (2013). A novel EPAC-specific inhibitor suppresses pancreatic cancer cell migration and invasion. Mol. Pharmacol. 83, 122–128 10.1124/mol.112.080689 23066090 PMC3533471

[b2] Calebiro D. Nikolaev V. O. Persani L. Lohse M. J. (2010). Signaling by internalized G-protein-coupled receptors. Trends Pharmacol. Sci. 31, 221–228 10.1016/j.tips.2010.02.002 20303186

[b3] Copsel S. Garcia C. Diez F. Vermeulem M. Baldi A. Bianciotti L. G. Russel F. G. Shayo C. Davio C. (2011). Multidrug resistance protein 4 (MRP4/ABCC4) regulates cAMP cellular levels and controls human leukemia cell proliferation and differentiation. J. Biol. Chem. 286, 6979–6988 10.1074/jbc.M110.166868 21205825 PMC3044954

[b4] Cupp M. E. Nayak S. K. Adem A. S. Thomsen W. J. (2013). Parathyroid hormone (PTH) and PTH-related peptide domains contributing to activation of different PTH receptor-mediated signaling pathways. J. Pharmacol. Exp. Ther. 345, 404–418 10.1124/jpet.112.199752 23516330

[b5] Dean T. Vilardaga J. P. Potts J. T. Jr and Gardella T. J. (2008). Altered selectivity of parathyroid hormone (PTH) and PTH-related protein (PTHrP) for distinct conformations of the PTH/PTHrP receptor. Mol. Endocrinol. 22, 156–166 10.1210/me.2007-0274 17872377 PMC2194631

[b6] Feinstein T. N. Wehbi V. L. Ardura J. A. Wheeler D. S. Ferrandon S. Gardella T. J. Vilardaga J. P. (2011). Retromer terminates the generation of cAMP by internalized PTH receptors. Nat. Chem. Biol. 7, 278–284 10.1038/nchembio.545 21445058 PMC3079799

[b7] Ferrandon S. Feinstein T. N. Castro M. Wang B. Bouley R. Potts J. T. Gardella T. J. Vilardaga J. P. (2009). Sustained cyclic AMP production by parathyroid hormone receptor endocytosis. Nat. Chem. Biol. 5, 734–742 10.1038/nchembio.206 19701185 PMC3032084

[b8] Fujimori A. Cheng S-L. Avioli L. V. Civitelli R. (1991). Dissociation of second messenger activation by parathyroid hormone fragments in osteosarcoma cells. Endocrinology 128, 3032–3039 10.1210/endo-128-6-3032 1645259

[b9] Gesty-Palmer D. Luttrell L. M. (2011). ‘Biasing’ the parathyroid hormone receptor: a novel anabolic approach to increasing bone mass? Br. J. Pharmacol. 164, 59–67 10.1111/j.1476-5381.2011.01450.x 21506957 PMC3171860

[b10] Gesty-Palmer D. Chen M. Reiter E. Ahn S. Nelson C. D. Wang S. Eckhardt A. E. Cowan C. L. Spurney R. F. Luttrell L. M. <(2006). Distinct β-arrestin- and G protein-dependent pathways for parathyroid hormone receptor-stimulated ERK1/2 activation. J. Biol. Chem. 281, 10856–10864 10.1074/jbc.M513380200 16492667

[b11] Gidon A. Al-Bataineh M. M. Jean-Alphonse F. G. Stevenson H. P. Watanabe T. Louet C. Khatri A. Calero G. Pastor-Soler N. M. Gardella T. J. <(2014). Endosomal GPCR signaling turned off by negative feedback actions of PKA and v-ATPase. Nat. Chem. Biol. 10, 707–709 10.1038/nchembio.1589 25064832 PMC4138287

[b12] Irannejad R. Tomshine J. C. Tomshine J. R. Chevalier M. Mahoney J. P. Steyaert J. Rasmussen S. G. Sunahara R. K. El-Samad H. Huang B. <(2013). Conformational biosensors reveal GPCR signalling from endosomes. Nature 495, 534–538 10.1038/nature12000 23515162 PMC3835555

[b13] Ito S. Suzuki S. Itoh T. (1993). Effects of a water-soluble forskolin derivative (NKH477) and a membrane-permeable cyclic AMP analogue on noradrenaline-induced Ca^2+^ mobilization in smooth muscle of rabbit mesenteric artery. Br. J. Pharmacol. 110, 1117–1125 10.1111/j.1476-5381.1993.tb13930.x 8298800 PMC2175778

[b14] Mahon M. J. (2012). The parathyroid hormone receptorsome and the potential for therapeutic intervention. Curr. Drug Targets 13, 116–128 10.2174/138945012798868416 21777186

[b15] Mannstadt M. Jüppner H. Gardella T. J. (1999). Receptors for PTH and PTHrP: their biological importance and functional properties. Am. J. Physiol. 277, F665–F675. 10564229 10.1152/ajprenal.1999.277.5.F665

[b16] Okazaki M. Ferrandon S. Vilardaga J. P. Bouxsein M. L. Potts J. T. Jr and Gardella T. J. (2008). Prolonged signaling at the parathyroid hormone receptor by peptide ligands targeted to a specific receptor conformation. Proc. Natl. Acad. Sci. USA 105, 16525–16530 10.1073/pnas.0808750105 18946036 PMC2571912

[b17] Pantazaka E. Taylor E. J. A. Bernard W. G. Taylor C. W. (2013). Ca^2+^ signals evoked by histamine H_1_ receptors are attenuated by activation of prostaglandin EP_2_ and EP_4_ receptors in human aortic smooth muscle cells. Br. J. Pharmacol. 169, 1624–1634 10.1111/bph.12239 23638853 PMC3724117

[b18] Potts J. T. Gardella T. J. (2007). Progress, paradox, and potential: parathyroid hormone research over five decades. Ann. N. Y. Acad. Sci. 1117, 196–208 10.1196/annals.1402.088 18056044

[b19] Rehmann H. (2013). Epac-inhibitors: facts and artefacts. Sci. Rep. 3, 3032 10.1038/srep03032 24149987 PMC3805970

[b20] Schmidt M. Evellin S. Weernink P. A. O. von Dorp F. Rehmann H. Lomasney J. W. Jakobs K. H. (2001). A new phospholipase-C-calcium signalling pathway mediated by cyclic AMP and a Rap GTPase. Nat. Cell Biol. 3, 1020–1024 10.1038/ncb1101-1020 11715024

[b21] Short A. D. Taylor C. W. (2000). Parathyroid hormone controls the size of the intracellular Ca^2+^ stores available to receptors linked to inositol trisphosphate formation. J. Biol. Chem. 275, 1807–1813 10.1074/jbc.275.3.1807 10636879

[b22] Takasu H. Gardella T. J. Luck M. D. Potts J. T. Jr and Bringhurst F. R. (1999). Amino-terminal modifications of human parathyroid hormone (PTH) selectively alter phospholipase C signaling via the type 1 PTH receptor: implications for design of signal-specific PTH ligands. Biochemistry 38, 13453–13460 10.1021/bi990437n 10521252

[b23] Taylor C. W. Tovey S. C. (2012). From parathyroid hormone to cytosolic Ca^2+^ signals. Biochem. Soc. Trans. 40, 147–152 10.1042/BST20110615 22260681

[b24] Tovey S. C. Taylor C. W. (2013). Cyclic AMP directs inositol (1,4,5)-trisphosphate-evoked Ca^2+^ signalling to different intracellular Ca^2+^ stores. J. Cell Sci. 126, 2305–2313 10.1242/jcs.126144 23525004 PMC3672942

[b25] Tovey S. C. Goraya T. A. Taylor C. W. (2003). Parathyroid hormone increases the sensitivity of inositol trisphosphate receptors by a mechanism that is independent of cyclic AMP. Br. J. Pharmacol. 138, 81–90 10.1038/sj.bjp.0705011 12522076 PMC1573637

[b26] Tovey S. C. Dedos S. G. Taylor C. W. (2006). Signalling from parathyroid hormone. Biochem. Soc. Trans. 34, 515–517 10.1042/BST0340515 16856848

[b27] Tovey S. C. Dedos S. G. Taylor E. J. A. Church J. E. Taylor C. W. (2008). Selective coupling of type 6 adenylyl cyclase with type 2 IP_3_ receptors mediates direct sensitization of IP_3_ receptors by cAMP. J. Cell Biol. 183, 297–311 10.1083/jcb.200803172 18936250 PMC2568025

[b28] Tovey S. C. Dedos S. G. Rahman T. Taylor E. J. A. Pantazaka E. Taylor C. W. (2010). Regulation of inositol 1,4,5-trisphosphate receptors by cAMP independent of cAMP-dependent protein kinase. J. Biol. Chem. 285, 12979–12989 10.1074/jbc.M109.096016 20189985 PMC2857138

[b29] van der Lee M. M. Verkaar F. Wat J. W. van Offenbeek J. Timmerman M. Voorneveld L. van Lith L. H. Zaman G. J. (2013). β-Arrestin-biased signaling of PTH analogs of the type 1 parathyroid hormone receptor. Cell. Signal. 25, 527–538 10.1016/j.cellsig.2012.11.012 23159578

[b30] Wang B. Bisello A. Yang Y. Romero G. G. Friedman P. A. (2007). NHERF1 regulates parathyroid hormone receptor membrane retention without affecting recycling. J. Biol. Chem. 282, 36214–36222 10.1074/jbc.M707263200 17884816

[b31] Wang B. Ardura J. A. Romero G. Yang Y. Hall R. A. Friedman P. A. (2010). Na/H exchanger regulatory factors control parathyroid hormone receptor signaling by facilitating differential activation of G(α) protein subunits. J. Biol. Chem. 285, 26976–26986 10.1074/jbc.M110.147785 20562104 PMC2930697

[b32] Wehbi V. L. Stevenson H. P. Feinstein T. N. Calero G. Romero G. Vilardaga J. P. (2013). Noncanonical GPCR signaling arising from a PTH receptor-arrestin-Gβγ complex. Proc. Natl. Acad. Sci. USA 110, 1530–1535 10.1073/pnas.1205756110 23297229 PMC3557057

[b33] Yamamoto T. Kambe F. Cao X. Lu X. Ishiguro N. Seo H. (2007). Parathyroid hormone activates phosphoinositide 3-kinase-Akt-Bad cascade in osteoblast-like cells. Bone 40, 354–359 10.1016/j.bone.2006.09.002 17046344

