# Supplementary material for: Sustained signalling by PTH modulates IP3 accumulation and IP3 receptors through cyclic AMP junctions
Source: J Cell Sci. 2015 Jan 15;128(2):408–20. doi: 10.1242/jcs.163071 (PMC4294780; doi:10.1242/jcs.163071)
Supplement: Supplementary Material [file supp_128_2_408__index.html]

Sustained signalling by PTH modulates IP3 accumulation and IP3 receptors through cyclic AMP junctions — Supplementary Material 

# Sustained signalling by PTH modulates IP3 accumulation and IP3 receptors through cyclic AMP junctions

## JCS163071 Supplementary Material

**Files in this Data Supplement:**

- **Supplementary Material**
