# Supplementary material for: Sustained signalling by PTH modulates IP3 accumulation and IP3 receptors through cyclic AMP junctions
Source: J Cell Sci. 2015 Jan 15;128(2):408–20. doi: 10.1242/jcs.163071 (PMC4294780; doi:10.1242/jcs.163071)
Supplement: Supplementary Material [file supp_128.2.408_JCS163071.pdf]

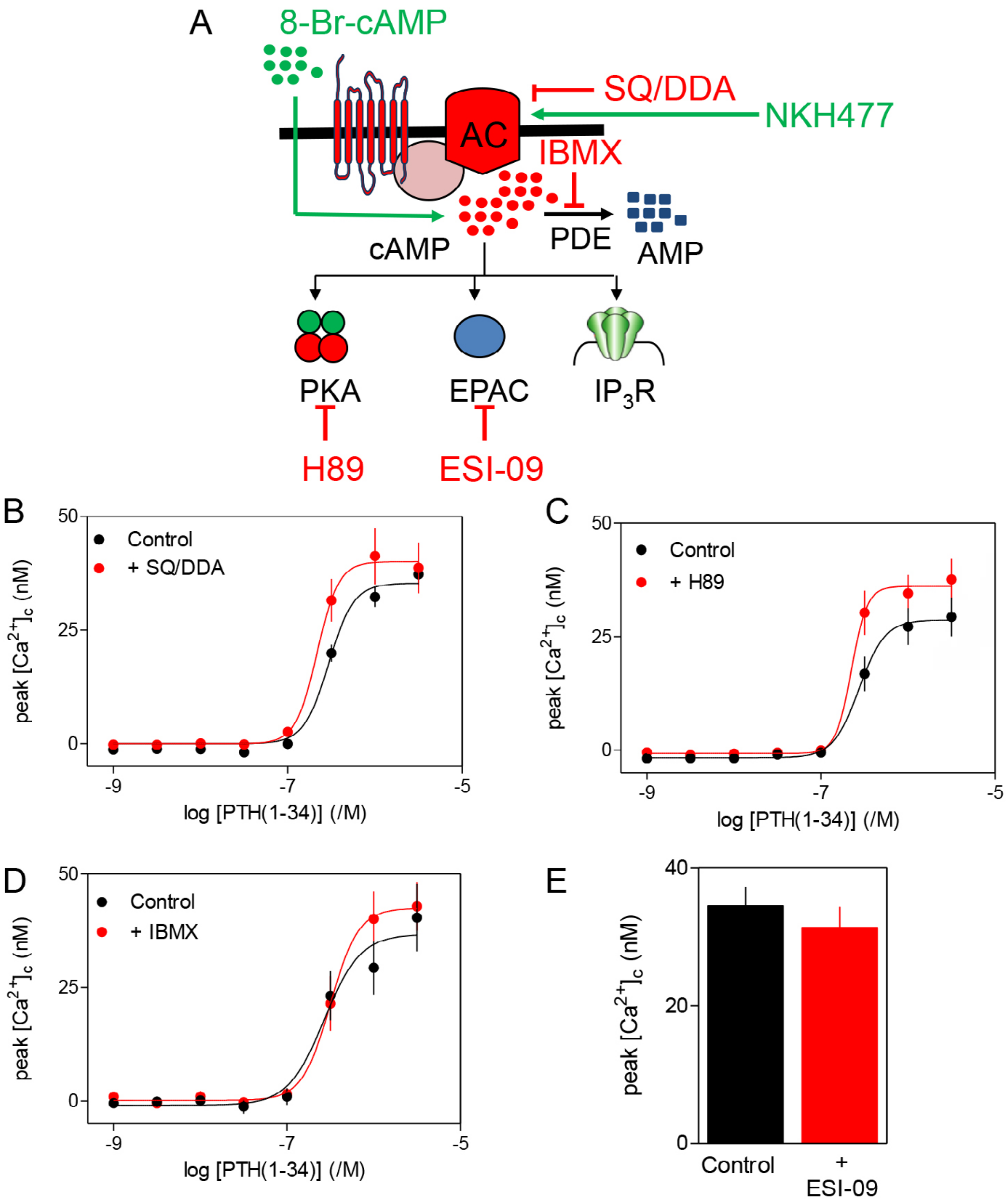

Fig. S1. Ca^{2+} signals evoked directly by high concentrations of PTH(1-34) are unaffected by inhibition of adenylyl cyclase, protein kinase A, cyclic nucleotide phosphodiesterases, or EPACs. (**A**) Targets of the inhibitors used. Cyclic AMP produced by adenylyl cyclase (AC) can stimulate protein kinase A (PKA), exchange proteins activated by cAMP (EPACs) or IP_3 receptors (IP_3R). DDA (2′,5′-dideoxyadenosine) and SQ 22536 inhibit adenylyl cyclase, while NKH477 directly stimulates it. IBMX inhibits the phosphodiesterases (PDE) that degrade cAMP. H89 inhibits PKA. ESI-09 is an antagonist of EPACs. 8-Br-cAMP is a membrane-permeant analogue of cAMP. (**B-D**) Cells were incubated with SQ22536 (1 mM) and DDA (200 µM) (B, SQ/DDA, 20 min) to inhibit adenylyl cyclase, H89 (C, 10 µM, 20 min) to inhibit protein kinase A, or IBMX (D, 1 mM, 5 min) to inhibit cyclic nucleotide phosphodiesterases before addition of the indicated concentrations of PTH(1-34). Results show the peak increases in $[Ca^{2+}]_c$ evoked by PTH(1-34). (**E**) Increases in $[Ca^{2+}]_c$ evoked by 3 µM PTH(1-34) alone or with the antagonist of EPACs, ESI-09 (10 µM, 5 min). Results (B-E) are means ± s.e.m., n = 3.

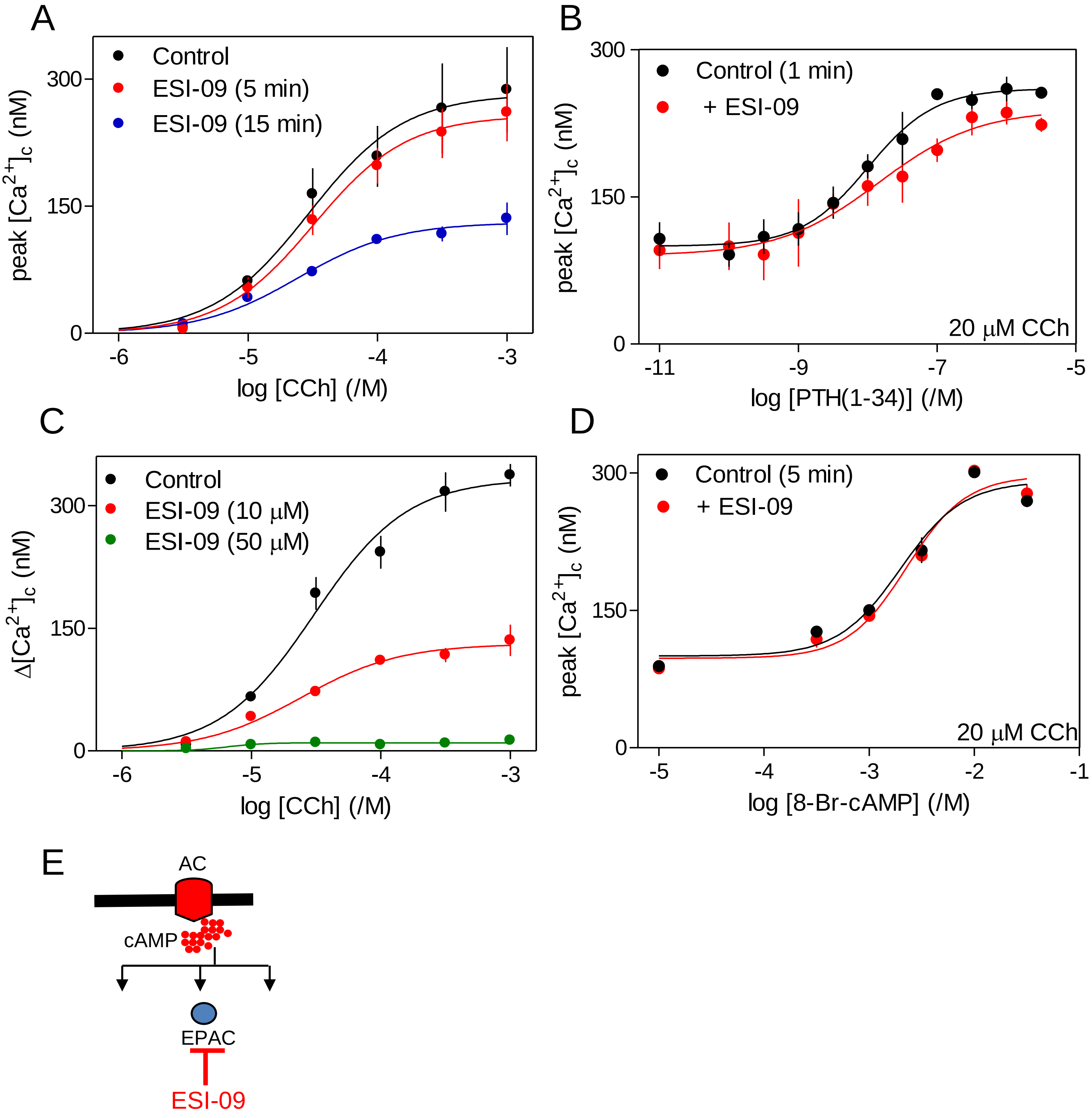

Fig. S2. Exchange proteins activated by cAMP (EPACs) do not mediate the effects of PTH(1-34) on carbachol-evoked Ca^{2+} signals. (**A**) HEK-PR1 cells were incubated with the EPAC antagonist, ESI-09 (10 μM for 5 or 15 min), before addition of the indicated concentrations of carbachol (CCh) in Ca^{2+}-free HBS. Results show the peak increase in $[Ca^{2+}]_c$ evoked by carbachol. (**B**) Similar analyses of cells treated with 10 μM ESI-09 for 5 min, and then stimulated with carbachol (20 μM) added 1 min after the indicated concentrations of PTH(1-34). (**C**) Experiments similar to those in A, show responses to carbachol after preincubation (15 min) with ESI-09 (0, 10 or 50 μM). (**D**) Effects of ESI-09 (10 μM, added 5 min before 8-Br-cAMP) on the Ca^{2+} signals evoked by carbachol after incubation with the indicated concentrations of the membrane-permeant analogue of cAMP, 8-Br-cAMP (5 min). Results (A-D) show means ± s.e.m., n = 3. (**E**) Target of the inhibitor used.

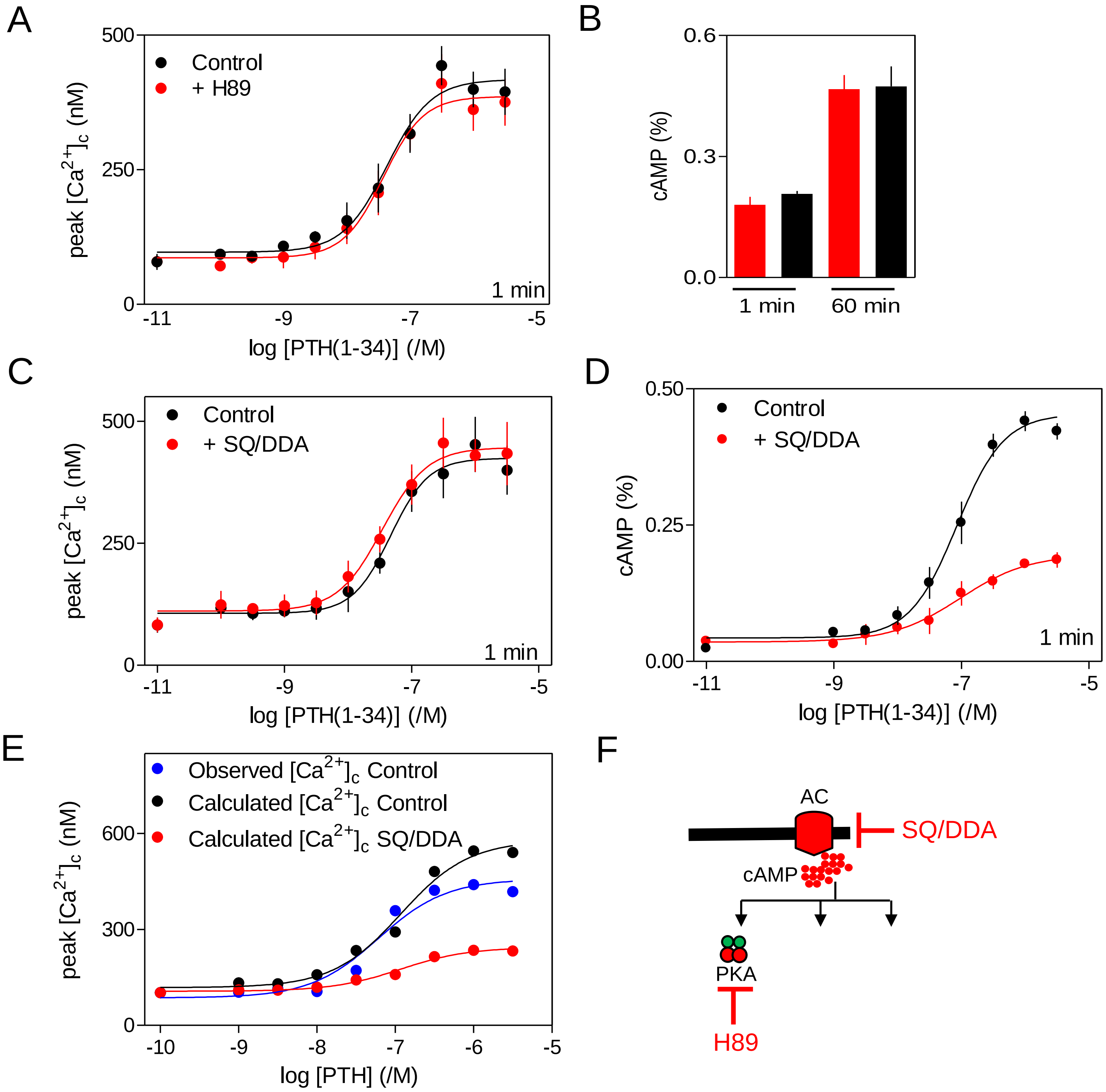

Fig. S3. Potentiation of carbachol-evoked Ca^{2+} signals by brief treatment with PTH(1-34) is unaffected by inhibition of protein kinase A or adenylyl cyclase. (**A, C**) Cells were incubated with H89 (A, 10 µM, 20 min, to inhibit PKA) or SQ22536 (1 mM) and DDA (200 µM) (C, SQ/DDA, 20 min, to inhibit AC) before addition of PTH(1-34) for 1 min and then carbachol (20 µM) in Ca^{2+}-free HBS. Results show the peak increase in $[Ca^{2+}]_c$ evoked by carbachol. **(B, D**) Effects of the same treatments on the increase in intracellular levels of cAMP (as percentages of ^{3}H-ATP, ^{3}H-ADP and ^{3}H-cAMP) evoked by PTH(1-34) (3 µM) for 1 min or 60 min (B) or by the indicated concentrations of PTH(1-34) for 1 min (D). Results (A-D) are means ± s.e.m. from at least 3 experiments. (**E**) The effect of inhibiting adenylyl cyclase on Ca^{2+} signals modulated by globally delivered cAMP would be readily detectable. Panel D demonstrates that SQ/DDA caused a 61% inhibition of the amount of cAMP produced in response to stimulation with PTH(1-34) for 1 min. Similar inhibition by SQ/DDA was observed after stimulation for 60 min (~70%, supplementary material Table S6). Comparison of responses with and without SQ/DDA allows computation of the concentrations of PTH(1-34) needed to stimulate production of equivalent amounts of cAMP with and without SQ/DDA. Assuming (for Ca^{2+} signals regulated by globally distributed cAMP) that the relationship between cAMP and $[Ca^{2+}]_c$ is unaffected by inhibition of AC (Fig. 5E, black line), we can predict the effects of SQ/DDA on potentiation of carbachol-evoked Ca^{2+} signals by PTH(1-34). The analysis has been performed for control cells (black line) and for cells treated with SQ/DDA (red), and compared with the observed control response (blue). The results demonstrate that if the effects of PTH(1-34) were mediated by a global cAMP signal, SQ/DDA would cause an easily resolved inhibition of the potentiated Ca^{2+} signals. (**F**) Targets of the inhibitors used.

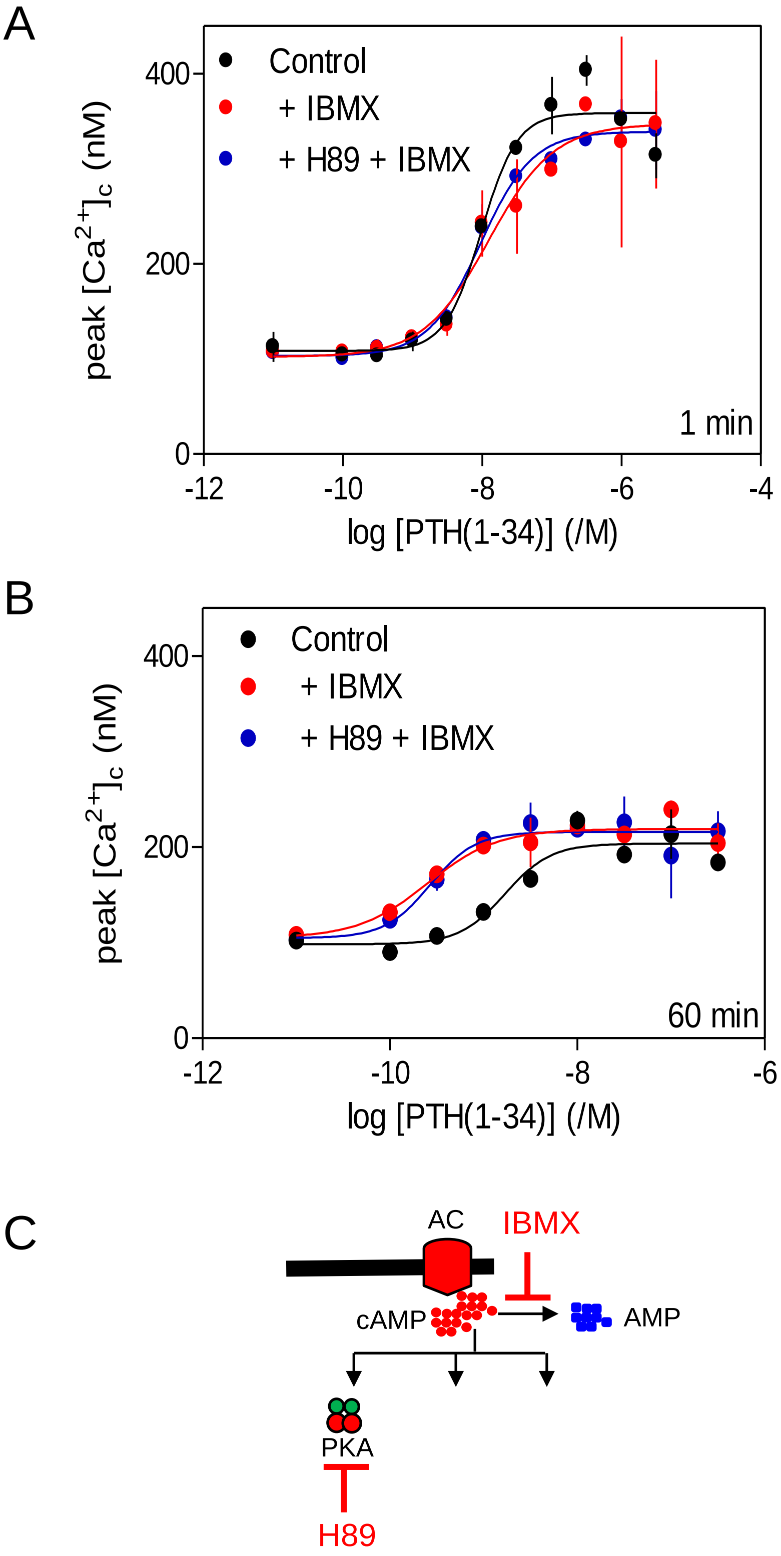

Fig. S4. Potentiation of carbachol-evoked Ca^{2+} signals by sustained stimulation with PTH(1-34) in the presence of IBMX is unaffected by inhibition of protein kinase A. (**A, B**) Cells were stimulated with PTH(1-34) for 1 min (A) or 60 min (B) before addition of carbachol (20 μM) in Ca^{2+}-free HBS. Cells were pretreated with IBMX (1 mM, 5 min, to inhibit cyclic nucleotide phosphodiesterases) alone or with H89 (10 μM, 20 min, to inhibit PKA) as indicated. Results (means ± s.e.m., n = 3) show the peak increase in $[Ca^{2+}]_c$ evoked by carbachol. (**C**) Targets of the inhibitors used.

Table S1. **Properties of the PTH analogues used.**

PTH(1-34)	SVSEIQLMHNLGKHLNSMERVEWLRKKLQDVHNF
PTH(2-38)	VSEIQLMHNLGKHLNSMERVEWLRKKLQDVHNFVALG
Tyr[1]PTH(1-34)	YVSEIQLMHNLGKHLNSMERVEWLRKKLQDVHNF
PTHrP(1-36)	AVSEHQLLHDKGKSIQDLRRRFFLHHLIAEIHTAQI
PTHrP(1-34)	AVSEHQLLHDKGKSIQDLRRRFFLHHLIAEIHTA
PTH(1-31)	SVSEIQLMHNLGKHLNSMERVEWLRKKLQDV

	Gs (AC)	Gq (PLC)	Signalling to AC from internalized PTH_1R	References
PTH(1-34)	Full agonist	Full agonist	Yes	Bisello et al., 2002; Castro et al., 2002; Cupp et al., 2013; Ferrandon et al., 2009; van der Lee et al., 2013.
PTHrP(1-36) PTHrP(1-34)	Full agonist	Full agonist	No[1]	Bisello et al., 2002; Cupp et al., 2013; Dean et al., 2008; Ferrandon et al., 2009.
PTH(2-38)	Full agonist	Almost Inactive	No	Cupp et al., 2013.
PTH(1-31)	Full agonist	Strong partial agonist	Yes	Cupp et al., 2013; Takasu et al., 1999.
PTH(3-34)[2]	Very weak partial agonist	Inactive	No	Takasu et al., 1999; Cupp et al., 2013; van der Lee et al., 2013.
Tyr[1]PTH(1-34)	Full agonist	Almost Inactive	Weak[3]	Cupp et al., 2013.

Sequences of the peptides used in this study are shown in the top panel using single-letter amino acid codes. The lower panel summarizes relevant properties of the PTH analogues. [1]PTHHrP(1-36) evokes internalization of PTH_1R, but not of functional AC signalling complexes. [2]Not used in this study, but included for comparison with earlier work (Tovey et al., 2008). [3]Tyr[1]PTH(1-34) is a weak partial agonist for receptor internalization; it is not known whether internalized AC signalling complexes accompany receptor internalization.

Table S2. Potentiation of carbachol-evoked Ca^{2+} signals by PTH(1-34).

	Carbachol 20 µM		Carbachol 1 mM	
	Maximal $\Delta[Ca^{2+}]_c$ (nM)	pEC_{50} (/M)	Maximal $\Delta[Ca^{2+}]_c$ (nM)	pEC_{50} (/M)
1 min	230 ± 10	7.5 ± 0.2	234 ± 29	7.8 ± 0.1
5 min	179 ± 37	7.1 ± 0.1	228 ± 9	7.6 ± 0.1
15 min	94 ± 7*	7.9 ± 0.1*	120 ± 7*	8.7 ± 0.1*
30 min	142 ± 38*	8.8 ± 0.1*	105 ± 30*	8.8 ± 0.1*
60 min	108 ± 7*	8.1 ± 0.2*	110 ± 16*	8.2 ± 0.2*

Experiments similar to those shown in Fig. 1B were used to establish the concentration-dependent effects of PTH(1-34) added for the indicated times before carbachol (20 µM or 1 mM) on carbachol-evoked Ca^{2+} signals. The EC_{50} (half-maximally effective concentration) for responses to carbachol alone was 32 µM, $pEC_{50} = 4.5 \pm 0.5$ (where $pEC_{50} = -\log EC_{50}$). Maximal $\Delta[Ca^{2+}]_c$ describes the difference between the peak Ca^{2+} signal evoked by carbachol alone and that evoked by carbachol in the presence of a maximally effective concentration of PTH(1-34). Results show means ± s.e.m., n = 4. $^*P < 0.05$ relative to paired comparisons at 1 min.

Table S3. Stimulation of cAMP accumulation and potentiation of carbachol-evoked Ca^{2+} signals by analogues of PTH.

	1 min				30 min			
	peak $[Ca^{2+}]_c$		cAMP		peak $[Ca^{2+}]_c$		cAMP	
	pEC_{50} (/M)	Maximal $\Delta[Ca^{2+}]_c$ (nM)	pEC_{50} (/M)	Maximal cAMP (%)	pEC_{50} (/M) [ΔpEC_{50}]	Maximal $\Delta[Ca^{2+}]_c$ (nM)	pEC_{50} (/M) [ΔpEC_{50}]	Maximal cAMP (%)
PTH(1-34)	7.7 ± 0.1	205 ± 13	7.0 ± 0.1	1.1 ± 0.1	8.8 ± 0.2* [1.1 ± 0.3]	105 ± 13*	7.5 ± 0.1* [0.5 ± 0.2]	1.9 ± 0.03*
Tyr[1]PTH(1-34)	6.7 ± 0.03	233 ± 10	6.1 ± 0.2	1.3 ± 0.2	7.4 ± 0.1* [0.7 ± 0.1]	148 ± 6*	6.7 ± 0.2* [0.6 ± 0.4]	2.6 ± 0.1*
PTHrP(1-36)	7.6 ± 0.1	281 ± 34	7.3 ± 0.1	0.2 ± 0.01	8.6 ± 0.2* [1.0 ± 0.3]	139 ± 2*	7.3 ± 0.2 [0.0 ± 0.3]	0.3 ± 0.01
PTH(2-38)	6.9 ± 0.1	216 ± 21	6.5 ± 0.04	0.7 ± 0.1	7.3 ± 0.1* [0.4 ± 0.2]	115 ± 13*	7.2 ± 0.2* [0.7 ± 0.2]	1.2 ± 0.1*

Cells were stimulated with PTH analogues for 1 or 30 min before measuring intracellular levels of cAMP (as percentages of ^{3}H-ATP + ^{3}H-ADP + ^{3}H-cAMP) and changes in $[Ca^{2+}]_c$ evoked by addition of carbachol (20 μM) in Ca^{2+}-free HBS. Results show maximal responses and pEC_{50} values as means ± s.e.m., n = 3. $\Delta[Ca^{2+}]_c$ is the difference between the peak Ca^{2+} signals evoked by carbachol alone or after treatment with the PTH analogue. Numbers in brackets show the difference in pEC_{50} value (where $pEC_{50} = -\log EC_{50}$) from measurements taken after incubation for 1 min or 30 min ($\Delta pEC_{50} = pEC_{50}^{1\,min} - pEC_{50}^{30\,min}$). $^{*}P < 0.05$ relative to paired comparisons at 1 min.

Table S4. Ca^{2+} signals evoked by carbachol after acute and sustained increases in intracellular cAMP.

	PTH(1-34)		NKH477		8-Br-cAMP	
	$\Delta[Ca^{2+}]_c$ (nM)	pEC_{50} (/M)	$\Delta[Ca^{2+}]_c$ (nM)	pEC_{50} (/M)	$\Delta[Ca^{2+}]_c$ (nM)	pEC_{50} (/M)
1 min	243 ± 16	7.5 ± 0.1	ND	ND	ND	ND
2 min	ND	ND	257 ± 3	5.0 ± 0.3	ND	ND
5 min	ND	ND	225 ± 9	5.0 ± 0.1	207 ± 7	3.5 ± 0.1
15 min	ND	ND	155 ± 10*	5.0 ± 0.1	ND	ND
30 min	127 ± 14*	8.7 ± 0.1*	124 ± 24*	6.0 ± 0.1*	113 ± 5*	3.7 ± 0.1

Increases in $[Ca^{2+}]_c$ evoked by 20 µM carbachol after incubations for the periods shown with PTH(1-34), NKH477 or 8-Br-cAMP. Results (means ± s.e.m., n = 3) show the maximal potentiated increase in $[Ca^{2+}]_c$ ($\Delta[Ca^{2+}]_c$) after subtraction of the response to carbachol alone (20 µM) and the sensitivity (pEC_{50}) for each stimulus. $^{*}P < 0.05$ relative to paired comparisons with the shortest incubation. ND, not determined.

Table S5. Effects of acute and sustained exposure to cAMP on carbachol-evoked Ca^{2+} signals.

	Peak $[Ca^{2+}]_c$ (nM)	
	1, 2 or 5 min	30 min
Carbachol alone	122 ± 6	113 ± 9
NKH477 then carbachol	331 ± 20	256 ± 12*
PTH(1-34) then carbachol	385 ± 26	268 ± 10*
8-Br-cAMP then carbachol	386 ± 21	222 ± 24*
8-Br-cAMP + NKH477 then carbachol	355 ± 27	257 ± 22*
NKH477 + PTH(1-34) then carbachol	356 ± 12	295 ± 25*
PTH(1-34) + 8-Br-cAMP then carbachol	360 ± 28	208 ± 8*

Effects of PTH(1-34) (1 µM, 1 or 30 min), NKH477 (200 µM, 2 or 30 min) and 8-Br-cAMP (10 mM, 5 or 30 min) alone or in combination on the peak increases in $[Ca^{2+}]_c$ evoked by carbachol (20 µM). Results are means ± s.e.m., n = 3. $^{*}P < 0.05$ relative to paired comparisons with the shortest period of stimulation.

Table S6. Substantial inhibition of adenylyl cyclase affects neither the acute nor sustained effects of PTH(1-34) on carbachol-evoked Ca^{2+} signals.

	1 min				60 min			
	peak $\Delta[Ca^{2+}]_c$		cAMP		peak $\Delta[Ca^{2+}]_c$		cAMP	
	pEC_{50} (/M)	Maximal $\Delta[Ca^{2+}]_c$ (nM)	pEC_{50} (/M)	Maximal ΔcAMP (%)	pEC_{50} (/M)	Maximal $\Delta[Ca^{2+}]_c$ (nM)	pEC_{50} (/M)	Maximal ΔcAMP (%)
Control	7.4 ± 0.2	364 ± 29	7.1 ± 0.1	0.40 ± 0.01	8.5 ± 0.20	153 ± 14	7.5 ± 0.01	0.6 ± 0.01
+ SQ/DDA	7.5 ± 0.1	341 ± 43	7.2 ± 0.5	0.16 ± 0.01*	8.9 ± 0.10	193 ± 32	7.9 ± 0.4	0.18 ± 0.03*
+ IBMX	7.7 ± 0.1	300 ± 26	7.4 ± 0.3	0.80 ± 0.1*	9.6 ± 0.1*	177 ± 18	7.7 ± 0.1	9.0 ± 0.02*
+ IBMX+SQ/DDA	7.8 ± 0.1	259 ± 8	ND	ND	9.2 ± 0.05*	178 ± 14	8.1 ± 0.05*	1.9 ± 0.2*

Cells were stimulated with PTH(1-34) for 1 min or 60 min alone or after preincubation with IBMX (1 mM, 5 min) to inhibit cyclic nucleotide phosphodiesterases, or SQ22536 (1 mM) and DDA (200 µM) (SQ/DDA, 20 min) to inhibit adenylyl cyclase. Intracellular levels of cAMP or peak $\Delta[Ca^{2+}]_c$ and their sensitivities to PTH(1-34) (pEC_{50}) are shown as means ± s.e.m., n = 3. $^{*}P < 0.05$ relative to paired time-matched comparisons without inhibitors.

Supplementary references

Bisello, A., Chorev, M., Rosenblatt, M., Monticelli, L., Mierke, D. F. and Ferrari, S. L. (2002). Selective ligand-induced stabilization of active and desensitized parathyroid hormone type 1 receptor conformations. *J. Biol. Chem.* **277**, 38524-38530.

Castro, M., Dicker, F., Vilardaga, J. P., Krasel, C., Bernhardt, M. and Lohse, M. J. (2002). Dual regulation of the parathyroid hormone (PTH)/PTH-related peptide receptor signaling by protein kinase C and β-arrestins. *Endocrinology* **143**, 3854-3865.

Cupp, M. E., Nayak, S. K., Adem, A. S. and Thomsen, W. J. (2013). Parathyroid hormone (PTH) and PTH-related peptide domains contributing to activation of different PTH receptor-mediated signaling pathways. *J. Pharmacol. Exp. Ther.* **345**, 404-418.

Dean, T., Vilardaga, J. P., Potts, J. T., Jr. and Gardella, T. J. (2008). Altered selectivity of parathyroid hormone (PTH) and PTH-related protein (PTHrP) for distinct conformations of the PTH/PTHrP receptor. *Mol. Endocrinol.* **22**, 156-166.

Ferrandon, S., Feinstein, T. N., Castro, M., Wang, B., Bouley, R., Potts, J. T., Gardella, T. J. and Vilardaga, J. P. (2009). Sustained cyclic AMP production by parathyroid hormone receptor endocytosis. *Nat. Chem. Biol.* **5**, 734-742.

Takasu, H., Gardella, T. J., Luck, M. D., Potts, J. T. and Bringhurst, F. R. (1999). Amino-terminal modifications of human parathyoid hormone (PTH) selectively alter phospholipase C signaling via the type 1 PTH receptor: Implications for design of signal-specific PTH ligands. *Biochemistry* **38**, 13453-13460.

Tovey, S. C., Dedos, S. G., Taylor, E. J. A., Church, J. E. and Taylor, C. W. (2008). Selective coupling of type 6 adenylyl cyclase with type 2 IP_3 receptors mediates a direct sensitization of IP_3 receptors by cAMP. *J. Cell Biol.* **183**, 297-311.

van der Lee, M. M., Verkaar, F., Wat, J. W., van Offenbeek, J., Timmerman, M., Voorneveld, L., van Lith, L. H. and Zaman, G. J. (2013). β-Arrestin-biased signaling of PTH analogs of the type 1 parathyroid hormone receptor. *Cell. Signal.* **25**, 527-538.
